# Fast-onset effects of *Pseudospondias microcarpa* (A. Rich) Engl. (Anacardiaceae) hydroethanolic leaf extract on behavioral alterations induced by chronic mild stress in mice

**DOI:** 10.1371/journal.pone.0278231

**Published:** 2023-02-02

**Authors:** Donatus Wewura Adongo, Priscilla Kolibea Mante, Kennedy Kwami Edem Kukuia, Charles Kwaku Benneh, Robert Peter Biney, Eric Boakye-Gyasi, Hilda Amekyeh, Benjamin Kingsley Harley, Augustine Tandoh, Prince Dagadu Okyere, Eric Woode

**Affiliations:** 1 Department of Pharmacology and Toxicology, School of Pharmacy, University of Health and Allied Sciences, Ho, Ghana; 2 Department of Pharmacology, Faculty of Pharmacy and Pharmaceutical Sciences, College of Health Sciences, Kwame Nkrumah University of Science and Technology, Kumasi, Ghana; 3 Department of Medical Pharmacology, University of Ghana Medical School, College of Health Sciences, University of Ghana, Korle Bu, Accra, Ghana; 4 Department of Pharmacology, School of Pharmacy and Pharmaceutical Sciences, University of Cape Coast, Cape Coast, Ghana; 5 Department of Pharmaceutics, School of Pharmacy, University of Health and Allied Sciences, Ho, Ghana; 6 Department of Pharmacognosy and Herbal Medicine, School of Pharmacy, University of Health and Allied Sciences, Ho, Ghana; McGill University Department of Psychiatry, CANADA

## Abstract

**Introduction:**

*Pseudospondias microcarpa* (Anacardiaceae) is a plant widely used traditionally for treating various central nervous system disorders. A previous study in our laboratory confirmed that the hydroethanolic leaf extract (PME) of the plant produces an antidepressant-like effect in rodent models of behavioral despair. However, its effect on depressive-like behavior induced by chronic mild stress (CMS) and its time course of action are still unknown. In this context, the long-term effects of PME on cognitive function and depressive- and anxiety-like behavior caused by CMS were assessed.

**Methods:**

Male ICR mice were exposed to CMS for nine weeks and anhedonia was evaluated by monitoring sucrose intake (SIT) weekly. PME (30, 100, or 300 mg kg^-1^) or fluoxetine (FLX) (3, 10, or 30 mg kg^-1^) was administered to the mice during the last six weeks of CMS. Behavioral tests—coat state, splash test, forced swimming test (FST), tail suspension test (TST), elevated plus maze (EPM), open field test (OFT), novelty suppressed feeding (NSF), EPM transfer latency, and Morris water maze (MWM)—were performed after the nine-week CMS period.

**Results:**

When the mice were exposed to CMS, their SIT and grooming behavior reduced (splash test), their coat status was poor, they became more immobile (FST and TST), more anxious (OFT, EPM, and NSF), and their cognitive function was compromised (EPM transfer latency and MWM tests). Chronic PME treatment, however, was able to counteract these effects. Additionally, following two (2) weeks of treatment, PME significantly boosted SIT in stressed mice (30 mg kg^-1^, *P*<0.05; 100 mg kg^-1^, *P*<0.05; and 300 mg kg^-1^, *P*<0.001), as compared to four (4) weeks of treatment with FLX.

**Conclusion:**

The present findings demonstrate that PME produces a rapid and sustained antidepressant-like action and reverses behavioral changes induced by chronic exposure to mild stressors.

## Introduction

Depression is a chronic mental illness primarily distinguished by gloomy mood and anhedonia; it is frequently accompanied by sleep problems, low self-esteem, guilty feelings, and suicide thoughts. Depression has significant direct and indirect costs for patients, their families, and society in addition to its direct impacts on health [1, 2]. In recent years, there has been mounting research that connects cognitive decline, anxiety, and depression [3, 4]. Cognitive impairment in depression affects a variety of cognitive functions, such as executive function, attention, memory, and psychomotor speed [3, 5]. The substantial co-morbidity between mood and anxiety disorders, as well as the intriguing overlap between the affective alterations seen in depression and anxiety disorders, point to a shared psychopathological pathway that may explain these changes [6].

The greatest environmental risk factor linked to the development of depression is stress exposure [7]. It has been established that exposure to stress, together with certain genetic risk factors, increases susceptibility to depression [8]. Some of the environmental elements that lead to the development of depressive disorders in humans have been hypothesized to be modeled by chronic mild stress (CMS) in mice [9, 10]. The CMS model simulates anhedonia, which is a primary symptom of depression and the distinguishing characteristic of melancholia. Anhedonia is a reduced ability to perceive pleasure [11]. The CMS model is predicated on the two hypotheses that sucrose solution consumption is a reliable indicator of reward sensitivity and that CMS has a generalized impact on reward sensitivity rather than having a specialized impact on responses to sweet tastes [12]. In the CMS paradigm, rats [13, 14] or mice [15, 16] are subjected to a variety of moderate stressors that change every few hours over the course of several weeks or months, such as overnight illumination, periods of food and/or water deprivation, cage tilt, and grouped housing. In repeated studies, it is common to note a decrease in the consumption and/or preference for a palatable mild (1–2%) sucrose solution as a measure of this procedure’s efficacy [17].

Several drugs have been shown to be effective in reversing CMS-induced anhedonia in rodents. These include tricyclic antidepressants [18, 19], selective serotonin reuptake inhibitors [1, 20], maprotiline, a particular inhibitor of noradrenaline reuptake [21], and moclobemide and brofaromine, inhibitors of monoamine oxidase [22, 23]. Chlordiazepoxide [21], *d*-amphetamine [23], and the neuroleptic agents chlorprothixene and haloperidol [23], on the other hand, were found to be ineffective in the CMS model. The aforementioned data thus implies that the CMS model only responds to antidepressant therapies.

Herbal medicines, with their high safety margins, have proven to be effective in the treatment of depression [24, 25]. Moreover, various reports have shown that drugs from plant sources are effective against the CMS model of depression [26, 27].

*Pseudospondias microcarpa* is one of such plants with medicinal properties. It is used as a sedative and for treating general central nervous system disorders [28]. In the forced swimming test (FST) and tail suspension test (TST), we have previously demonstrated that the hydroethanolic leaf extract of the plant (PME) exerts antidepressant-like effects by interacting with the 5-HT system, nitric oxide pathway, and the glycine/N-methyl-D-aspartate (NMDA) receptor complex [29]. Additionally, it produces a rapid and sustained antidepressant-like effect in the repeated open-space swim model while improving cognitive function [30].

In the current study, we use the CMS paradigm, a paradigm with greater face and construct validity for clinical depression, to confirm in mice the great potential of PME in preventing or managing depression. Additionally, we evaluate the effect of PME on depression-related cognitive impairment and co-morbid anxiety.

## Materials and methods

### Plant collection and extract preparation

Fresh *P*. *microcarpa* leaves were obtained at the Kwame Nkrumah University of Science and Technology (KNUST) campus in Kumasi, Ghana (6° 40.626’N, 1° 34.041’W), and they were authenticated at the Department of Herbal Medicine, Faculty of Pharmacy and Pharmaceutical Sciences, KNUST, Kumasi. The plant’s leaves were ground into a fine powder after being air dried for seven days. Over the course of 72 hours, the powder was cold percolated with 70% (v/v) ethanol in water. The resulting extract was then condensed into a syrupy mass under reduced pressure at 60°C in a rotary evaporator. It was then preserved in a refrigerator for usage after being further dried for a week at 50°C in a hot air oven. The yield was 20.5% (w/w). In this study, the crude extract is referred to as PME or extract.

### Fourier-transform infrared spectroscopy (FT-IR) analysis of crude extract

To characterize the extract and identify the possible functional groups that may be present in the sample, triplicate FT-IR (PerkinElmer UATR Two) spectra over a range of 400–4000 cm^−1^ were generated and the baseline corrected. This spectral region is unique for every compound/compound mixture and hence can be used for identification and quality control of the extract used in this study.

### Animals

Male ICR mice (weighing 20–25 g, aged 8 weeks) were bought from the Noguchi Memorial Institute for Medical Research in Accra, Ghana, and housed in an animal facility at the Department of Pharmacology at KNUST in Kumasi, Ghana. The animals were kept in groups of five in cages made of stainless steel that measured 34 cm by 47 cm by 18 cm. Soft wood shavings were used as bedding, and the animals were kept in cages under controlled conditions that included a 12/12-hour light-dark cycle (light cycle: 06:00 to 18:00, dark cycle: 18:00–06:00) and a temperature of 24 to 25°C. All mice had free access to food (commercial pellet diet; Agricare, Kumasi, Ghana) and water. A period of two weeks was allowed for acclimatization to the laboratory environment. All laboratory procedures were conducted in accordance with accepted principles for laboratory animal use and care [31]. The Faculty Ethics Committee, Faculty of Pharmacy and Pharmaceutical Sciences, KNUST, gave its approval for the study.

### Test drug solutions

Fluoxetine hydrochloride (Prozac^®^, FLX) was purchased from Eli Lilly and Company Ltd. (Basingstoke, England). Test samples were freshly prepared every day in saline (NaCl, 0.9%) and administered *p*.*o*. in a volume of 10 mL kg^-1^ at the doses stated. Vehicle or control animals received 0.9% saline in a volume of 10 mL kg^-1^. Administration of test compounds was done *via* an oral gavage.

### CMS and sucrose intake (SIT)

The CMS protocol used in this study was adopted from the procedure described by Papp, Gruca (18). Mice were housed individually in small cages (8 cm × 13.5 cm × 8 cm) and trained for two weeks to consume a palatable sucrose solution contained in 150 mL water bottles placed above the cages. A 14-hour period of no food or water was followed by six 1-hour tests in which 1% sucrose in water was made available. At the conclusion of each test, SIT was determined by weighing the pre-weighed bottles containing the solution and calculating the change in weights. Testing began at 10:00 am.

The animals were randomly split into two matched groups (CMS and non-stressed [NS] groups) after the last baseline test, and were housed individually as previously mentioned in two different rooms. Subsequently, SIT was similarly measured at weekly intervals for the duration of the experiment.

**Fig 1:**
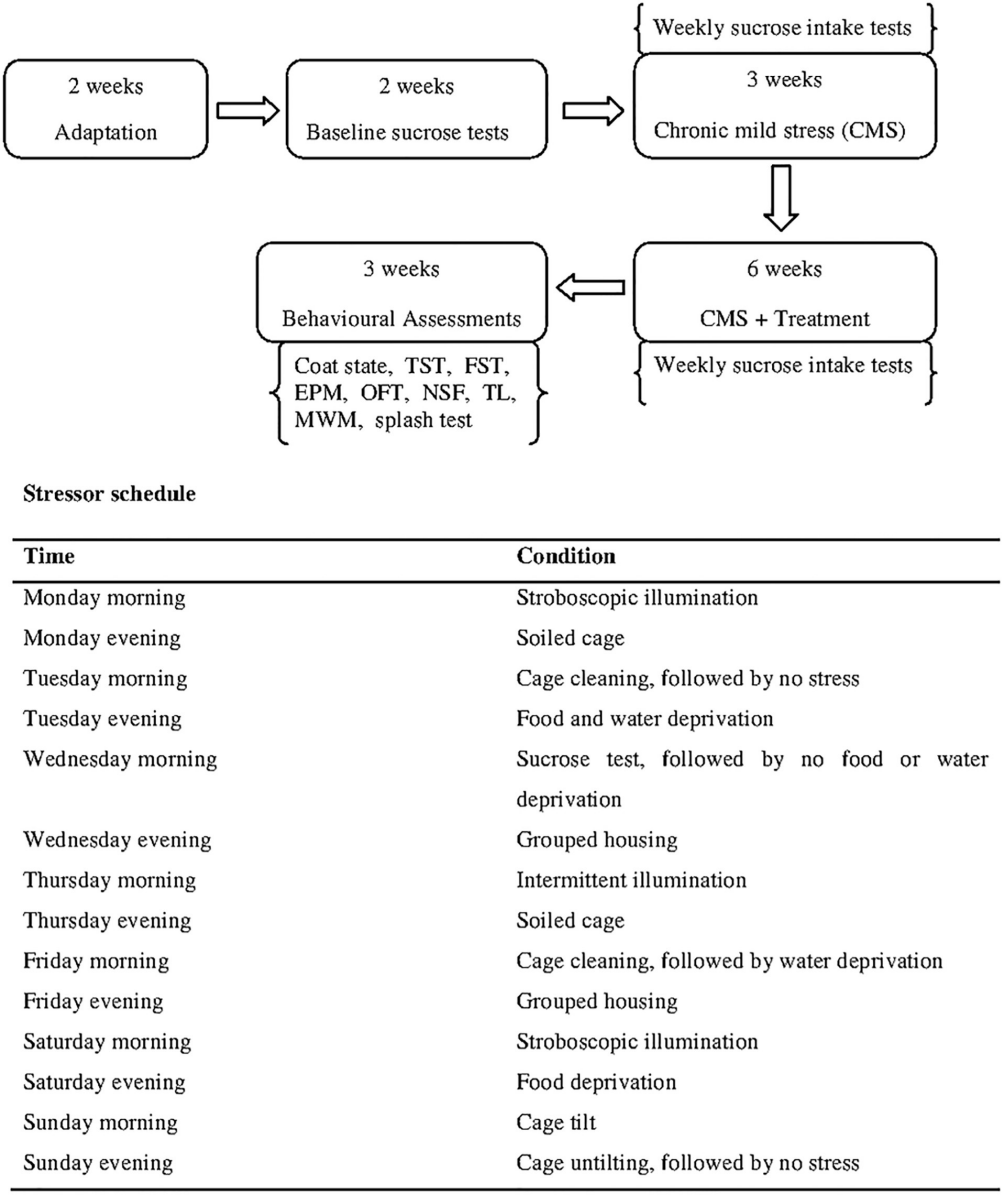
Schematic diagram showing the experimental design of the chronic mild stress (CMS) procedure.

The CMS group of animals was subjected to the CMS procedure. A breakdown of the stress regimen for each week is as follows: two periods of deprivation of food or water, two periods of 45° cage tilt, two periods of intermittent illumination (lights on and off every 2 h), two periods in a soiled cage (250 mL water in sawdust bedding), two periods of paired housing, two periods of low-intensity stroboscopic illumination (150 flashes/min), and two periods of no stress. According to the schedule shown in Fig 1, each stressor was used for 10 to 14 hours, continuously, day and night.

The other group representing NS animals was housed in a separate room and had no contact with the stressed animals. They were only deprived of food and water for 14 h before each sucrose test.

Both CMS (stressed) and NS animals were then separated into matched subgroups (n = 8 mice per group) based on SIT after the initial three weeks of stress. For the subsequent six weeks, they received daily oral administration of normal saline (10 mL kg^-1^), PME (30, 100, or 300 mg kg^-1^), or FLX (3, 10, or 30 mg kg^-1^) at 10:00 am.

Groupings were as follows:

NS groups: non-stressed control, PME (30, 100, or 300 mg kg^-1^), and FLX (3, 10, or 30 mg kg^-1^).
CMS groups: CMS control, PME (30, 100, or 300 mg kg^-1^), and FLX (3, 10, or 30 mg kg^-1^).

### Weight variation

Before and during the unpredictable CMS periods, the body weights of the animals were recorded every Monday at 8:00 am.

### Behavioral assessments

Following the CMS procedure (that is, after week 6 of treatment), a number of behavioral tests were conducted to evaluate depression (TST, FST, splash test, coat state assessment), anxiety (elevated plus maze [EPM], novelty suppressed feeding [NSF], open field test [OFT]), and cognitive function (Morris water maze [MWM] task, and EPM transfer latency test). Tests were performed in the following order over 3 weeks: coat state assessment (day 1), splash test (day 2), TST (day 4), FST (day 6), OFT (day 8), NSF (day 11), MWM task (days 13−18), and EPM transfer latency (days 20 and 21). Tests were performed from 9:00 am to 1:00 pm and no stressors were applied before each test.

#### Coat state assessment

Each animal was carefully removed from its home cage for this test, and a blinded experimenter examined and methodically recorded the condition of the coat at eight different body parts, including the head, neck, forepaws, dorsal and ventral regions, hind legs, tail, and genital region [32]. For each body area, a score of 0 was given for a coat in good form, and a score of 1 for a dirty or disheveled coat. The total score for all the body areas assessed was calculated and used as the final score.

#### Splash test

To analyze the grooming behaviors of both stressed and NS mice, this test was run a day following the coat state assessment. Mice were splashed with a 10% sucrose solution in their home cages, and for five minutes, the animals were videotaped. The frequency of grooming was calculated as the total number of licks during the 5-minute interval. Nose/face grooming (strokes along the snout), head washing (semi-circular movements over the top of the head and behind the ears), and body grooming (body fur licking) were among the grooming episodes that were observed [33].

#### Tail suspension test

This test was performed based on the method described by Steru, Chermat [34]. Briefly, each mouse was separately suspended by the tail from a horizontal ring-stand bar 50 cm above the floor using adhesive tape positioned 1 cm from the tip of the tail. The posture of the mice was such that the horizontal plane was parallel to the base of their tails. A 6-minute videotape was made of each test session. The final four minutes of the six-minute period’s behaviors were then analyzed with a public domain software JWatcher, version 1.0 (University of California, Los Angeles, USA, and Macquarie University, Sydney, Australia) for mobility and immobility durations. A mouse was considered to be immobile when it stopped struggling and remained suspended on the horizontal bar.

#### Forced swim test

This experiment was performed according to the procedure described by Porsolt, Bertin [35] with modifications. Briefly, mice were forced to swim for 6 min in polypropylene cylinders with a 10 cm diameter and a height of 25 cm that contained 10 cm of water at 25°C. Using a camcorder, the final four minutes of the six-minute test session were recorded, and the duration of immobility—which represents the condition of depression—was examined as previously reported for the TST. When a mouse ceased actively moving to stay afloat and keep its head above water, it was regarded as immobile.

#### Open field test

This test was performed in control and CMS mice to study exploratory and anxiety behavior [36]. The open field apparatus used consisted of a white Plexiglas box (50 cm × 50 cm × 20 cm) with its floor divided into 16 squares with black lines. The center was designated as four squares, and the periphery as 12 squares along the walls. A 45 W low intensity diffuse light was used to illuminate the equipment from 45 cm above the ground. The experiment was conducted in a darkened room with the exception of the open field. The animals were each placed in the center square and observed for five minutes. Then, videotaping and later analysis were done on the center activity (number and duration of entries into the central squares with all four paws) and number and duration of rearing (animal standing upright on its hind limbs). The device was cleaned with 5% alcohol in between tests.

#### Elevated plus maze test

This test assesses unconditioned anxiety-like behavior in rats and mice [37]. The EPM consisted of two open arms (30 cm × 5 cm), two enclosed arms (30 cm × 5 cm), and a connecting central platform (5 cm × 5 cm). The maze was elevated 38.5 cm above the ground. Each mouse was positioned in the central neutral zone, facing one of the close arms, at the start of the five-minute session, and their behavior was recorded on camera. The videos were then scored based on behavioral indicators such as the number of entries and the time spent in the open arms using the public domain software JWatcher^™^. An arm entry was defined as a mouse having entered an arm of the maze with all four legs.

#### Novelty suppressed feeding test

The test was carried out during a 5-min period as previously described [38]. The testing device was a plastic box (50 cm x 50 cm x 20 cm) with 2 cm of wooden bedding covering the bottom. The animals were denied access to any food in the home cage for the previous twenty-four hours before behavioral testing. During testing, a solitary food pellet was put on a white paper platform that was positioned in the middle of the box. The test began after the animal was placed in a corner of the box. The latency to feed (the length of time before mice began to eat) was manually tracked.

#### Morris water maze test

Additionally assessed were the impacts of mouse behavior on hippocampal-dependent spatial learning and memory *via* the MWM task [39]. The MWM apparatus was a tank with dimensions of 120 cm in diameter and 60 cm in height, filled with water to a depth of 45 cm, and kept at a temperature of 23±1°C. In order to make the water opaque, non-toxic black ink was added. By two imaginary perpendicular lines crossing the middle of the tank, the tank was divided into four equal quadrants (NE, SE, NW, and SW). To allow a mouse to quickly climb and escape from the water, a moveable, circular, black platform with a diameter of 5 cm was positioned in the center of the SW quadrant (the target quadrant) and lowered 2 cm below the water’s surface. Each session was recorded with a video camera approximately 100 cm above the center of the maze. The environment was kept lightless to maintain visual extra-maze cues and minimize noise disturbance.

Place navigation and a spatial probe trial made up the MWM task. Animals in the place navigation test underwent four training trials lasting two minutes each over the course of five days. It assessed motivation and ability to swim and escape from the aversive situation of being placed in the water by associating the platform with escape. The platform was in the middle of the SW quadrant for each trial. Every time, the mouse entered the pool facing the wall from a different starting position, altering the straight path to the platform. Briefly, the location of the platform remained constant and mice were allowed to swim for 60 s or until they located the platform. Mice were manually led to the platform after failing to find it within 60 seconds, where they remained for at least 5 seconds before being returned back to their cage.

Mice underwent the probe trial in which the platform was removed 24 hours following the final training trial of the escape acquisition test. The time spent in the target quadrant, or the quadrant where the platform was throughout the training sessions, was calculated as a measure of spatial memory during the 60-second probing trial.

#### EPM transfer latency

The procedure for assessing learning and memory was performed as previously described [40, 41]. Evaluating learning and memory in mice was done using the exteroceptive behavioral model (EPM), in which the stimulus was external to the body. Each mouse was positioned at one end of the outstretched arms on the first day, facing away from the central platform. The amount of time a mouse needs to move into one of the closed arms with all four of its legs is known as transfer latency. For each animal, this was noted on the first day. Animals were gently pushed into one of the two closed arms and transfer latency was calculated as 90 seconds if they did not enter one of the closed arms within that time frame. Each mouse was allowed to explore the maze for another 2 min and returned to its home cage. Retention of this learned task (memory) was examined 24 h after the first day of trial.

### Statistical analysis

A sample size of eight animals was used in all tests. All data are presented as mean ± standard error of the mean (SEM). To compare differences between groups, one-way analysis of variance (ANOVA) was performed with Newman-Keuls test as *post hoc*. Two‑way ANOVA with Bonferroni’s *post hoc* test (*treatment × dose*) was also performed to compare the NS and CMS groups. Time-course curves were subjected to two-way (*treatment × time*) repeated measures ANOVA with Bonferroni’s *post hoc* test. All statistical evaluations were performed using GraphPad Prism for Windows 5 (GraphPad Software, San Diego, CA, USA). Bonferroni’s test or Newman-Keuls test of *P* values < 0.05 were regarded as statistically significant.

## Results

### FT-IR analysis

**Fig 2:**
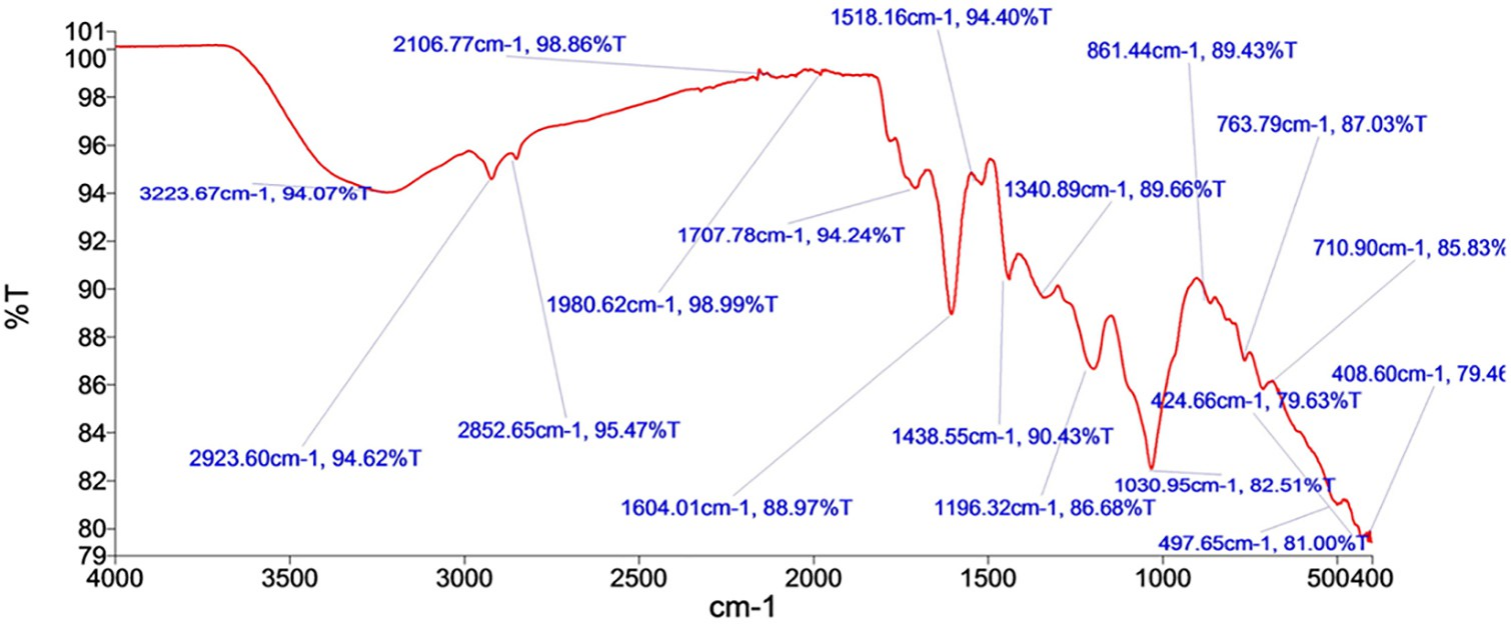
Infrared spectrum of the hydroethanolic leaf extract of *P*. *microcarpa* (PME).

FT-IR spectroscopy was used for distinct functional group identification over an IR region of 400–4000 cm^-1^ (Fig 2). Characteristic spectra in the region were used as the fingerprint spectra for subsequent comparison of extracts. Baseline corrected IR spectra and peak values are provided in the Supporting information.

### Sucrose intake

From the initial baseline test, all animals drank approximately 1.68–3.72 g of sucrose solution (**data captured under supplementary materials**). Exposure of mice to various stressors in the CMS procedure for three weeks (week 0) resulted in a significant decrease in the consumption of 1% sucrose solution as compared to the observation in the NS group (*P*<0.001). SIT by the NS mice however remained at the same level (approximately 2.60 g). The difference between NS and CMS animals treated with vehicle persisted at a similar level for the remainder of the 6-week treatment period (*P*<0.001).

**Fig 3:**
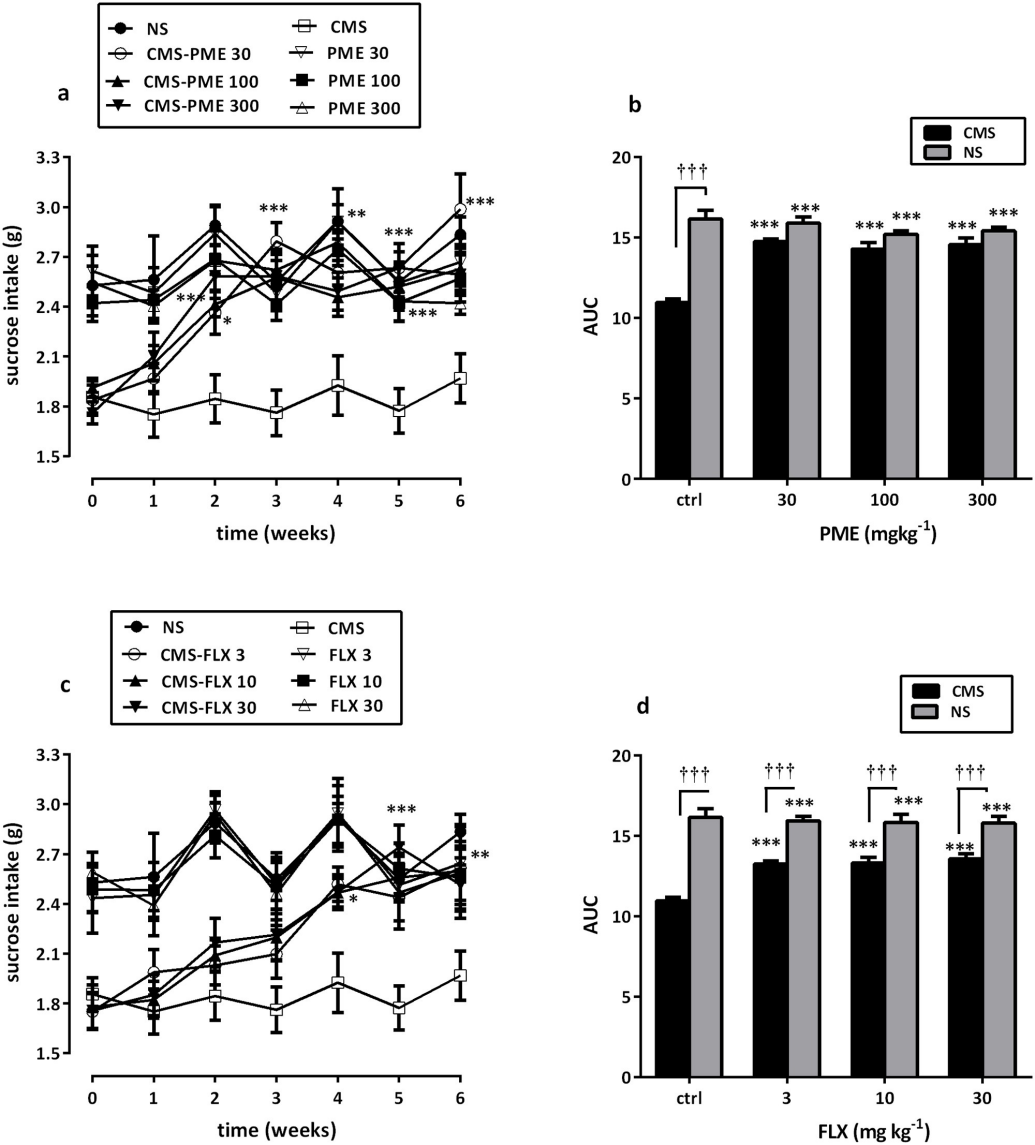
SITs of NS and CMS mice treated with saline, PME (30–300 mg kg^-1^), or FLX (3–30 mg kg^-1^). Data are presented as time-course curves (a and c) and as mean±SEM (n = 8) of the AUCs (b and d). Significantly different from CMS control: *P<0.05, **P<0.01, ***P<0.001 (Newman-Keuls’ *post hoc* test for the AUC or two-way ANOVA followed by Bonferroni’s *post hoc* test for time-course curves). Statistically significant difference when CMS and NS groups were compared: †††*P*<0.001 (two-way repeated measures ANOVA followed by Bonferroni’s *post hoc* test). ctrl, control.

Chronic treatment with PME and FLX had no significant effect on SIT in NS animals (*P*<0.05). However, as shown in Fig 3a, PME increased sucrose consumption in the stressed (CMS) animals, resulting in significant effects of treatment (*F*_3,196_ = 34.48, *P*<0.0001), interaction (*F*_18,196_ = 2.160, *P* = 0.0054), and time (*F*_6,196_ = 16.17, *P*<0.0001). Compared to week 0 scores, increases in SIT in stressed animals treated with PME reached statistical significance after 2 weeks of treatment as shown in Fig 3a (30 mg kg^-1^, *P*<0.05; 100 mg kg^-1^, *P*<0.05; and 300 mg kg^-1^, *P*<0.001). This effect was maintained for the remainder of the treatment period. Furthermore, by week 3, sufficient recovery from the stress-induced deficit in sucrose consumption was observed as compared to the vehicle-treated control (NS) mice. Analysis of the areas under the curves (AUCs) by one-way ANOVA demonstrated a significant increase in SIT (*F*_3,28_ = 32.29, *P*<0.0001; Fig 3b) for PME, with the Newman-Keuls *post hoc* analysis giving statistically significant data at all the doses tested (all *P*<0.001).

In the CMS animals, FLX (Fig 3c) increased sucrose consumption, resulting in significant effects of treatment (*F*_3,196_ = 13.40, *P*<0.0001) and time (*F*_6,196_ = 13.98, *P*<0.0001). However, unlike PME, the increased sucrose consumption in the stress animals reached significance after 4 weeks of FLX treatment (*P*<0.05 in each instance). In analyzing the AUC for FLX-treated CMS animals, one-way ANOVA demonstrated a significant increase in SIT (*F*_3,28_ = 19.31, *P*<0.0001; Fig 3d) as compared to the data for the CMS control mice.

### Weight change

**Fig 4:**
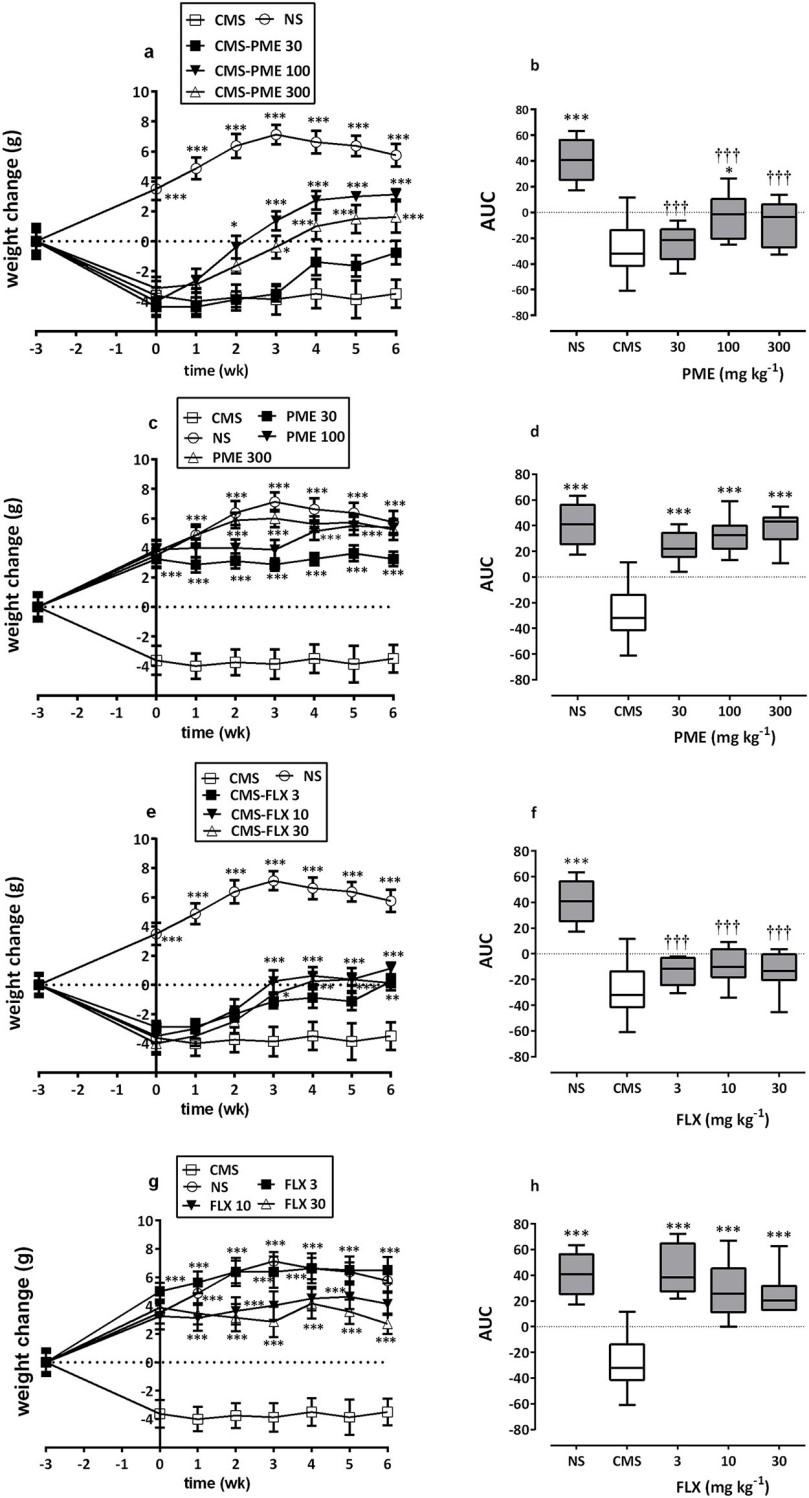
Effects of PME (30–300 mg kg^-1^) and FLX (3–30 mg kg^-1^) on weight change in NS and CMS mice. Data are presented as time course curves (a, c, e, and g) and as mean±SEM (n = 8) of AUCs (b, d, f, and h). Significantly different from stress-control group: *P<0.05, ***P<0.001 (Newman-Keuls *post hoc* test for the AUCs or two-way ANOVA followed by Bonferroni’s *post hoc* test for time-course curves). Significant difference when compared to NS-control mice: †††*P*<0.001 (one-way ANOVA followed by Newman-Keuls’ *post hoc* test).

Weight change in saline-control mice increased gradually until it peaked in the third week. Mice subjected to the CMS paradigm for 3 weeks (week 0) showed a significant decrease in weight change relative to the control (NS) group (*P*<0.001). Furthermore, the extract-treated stressed animals showed a significant increase in body weight (*F*_4,280_ = 136.7, *P*<0.0001 as compared to the CMS-saline group; Fig 4a), with the Bonferroni’s *post hoc* analysis showing significant differences after weeks 2 and 3 for the 100 and 300 mg kg^-1^ doses, respectively (*P*<0.05 in each case). FLX also increased weight change (*F*_4,280_ = 153.6, *P*<0.0001; Fig 4e) in stressed mice, reaching a significantly different value after week 3 (*P*<0.001 for 10 mg kg^-1^ and *P*<0.05 for 30 mg kg^-1^) and week 6 (*P*<0.01 for 3 mg kg^-1^).

### Coat state

**Fig 5:**
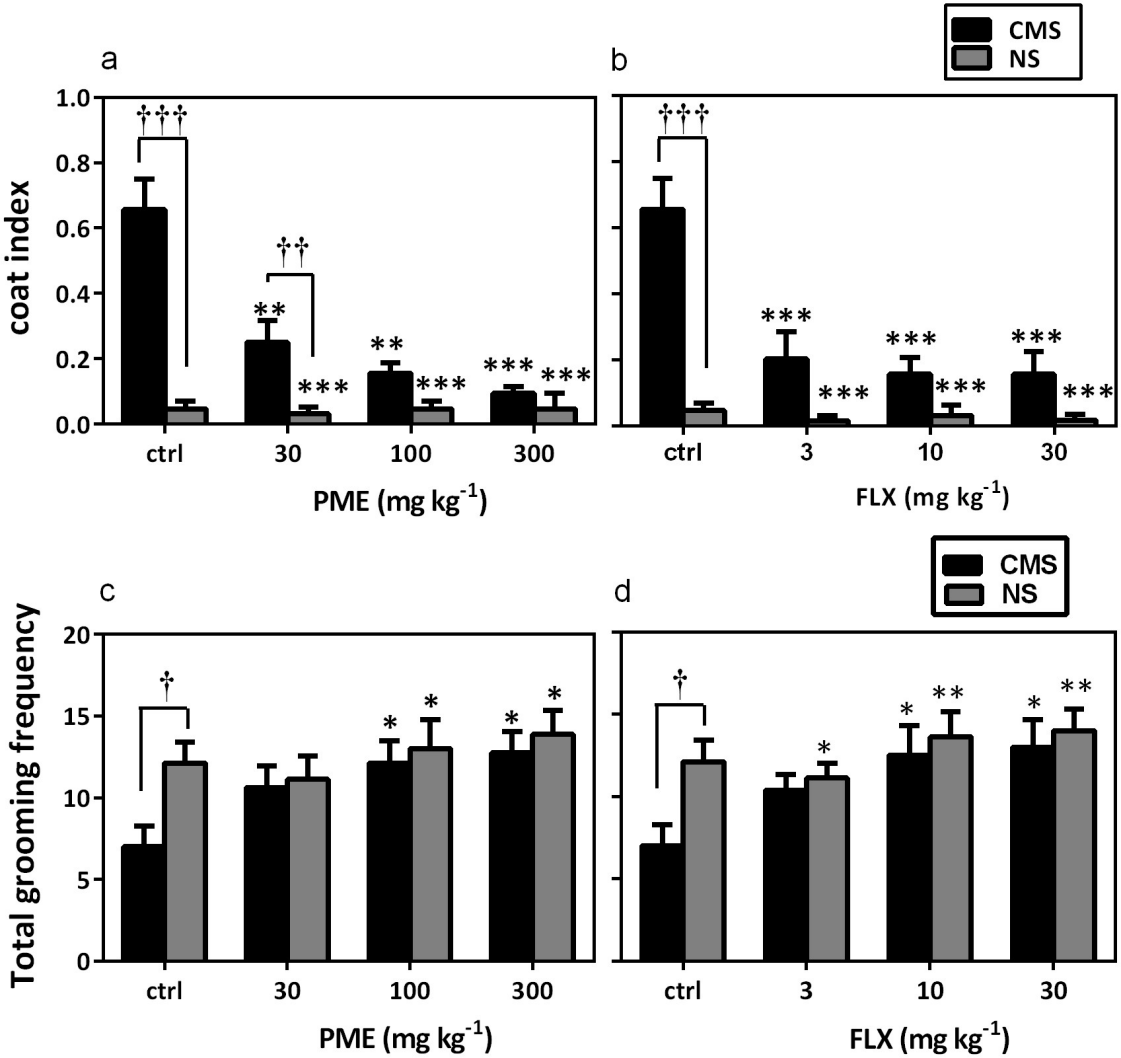
Effects of PME (30–300 mg kg^-1^) and FLX (3–30 mg kg^-1^) on coat index (a and b) and total frequency grooming frequency (c and d) in the coat state assessment and splash test, respectively. Data are expressed as group mean±SEM (n = 8). Significantly different from CMS-control group: *P<0.05, **P<0.01, ***P<0.001 (one-way ANOVA followed by Newman-Keuls *post hoc* test) and significant difference when CMS and NS groups were compared: †*P*<0.05, ††*P*<0.01, †††*P*<0.001 (two-way repeated measures ANOVA followed by Bonferroni’s *post hoc* test). ctrl, control.

The coat states of both stressed and NS mice were scored nine weeks after the beginning of the unpredictable CMS regimen. As shown in Fig 5, a significant difference in coat state was observed between NS and CMS-control mice at the end of the unpredictable CMS regimen (*P*<0.001). PME (F_4,35_ = 19.70, *P*<0.0001; Fig 5a) and FLX (F_4,35_ = 12.01, *P*<0.0001; Fig 5b) significantly reversed the degradation of coat state induced by CMS in stressed mice. In addition, no significant difference in coat state was observed between NS mice treated with test compounds and the NS-control group (*P*>0.05).

### Splash test

Total grooming frequency showed a significant difference between NS control and stressed-control groups at the end of the unpredictable CMS regimen (*P*<0.05). Treatment of stressed mice with PME (*F*_4,35_ = 3.145, *P* = 0.026; Fig 5c) and FLX (*F*_4,35_ = 2.891, *P* = 0.036; Fig 5d) significantly increased the total grooming frequency in the splash test when compared to the CMS group. No significant difference was observed between drug-treated control mice and the NS-control mice (*P*>0.05).

### Tail suspension test

**Fig 6:**
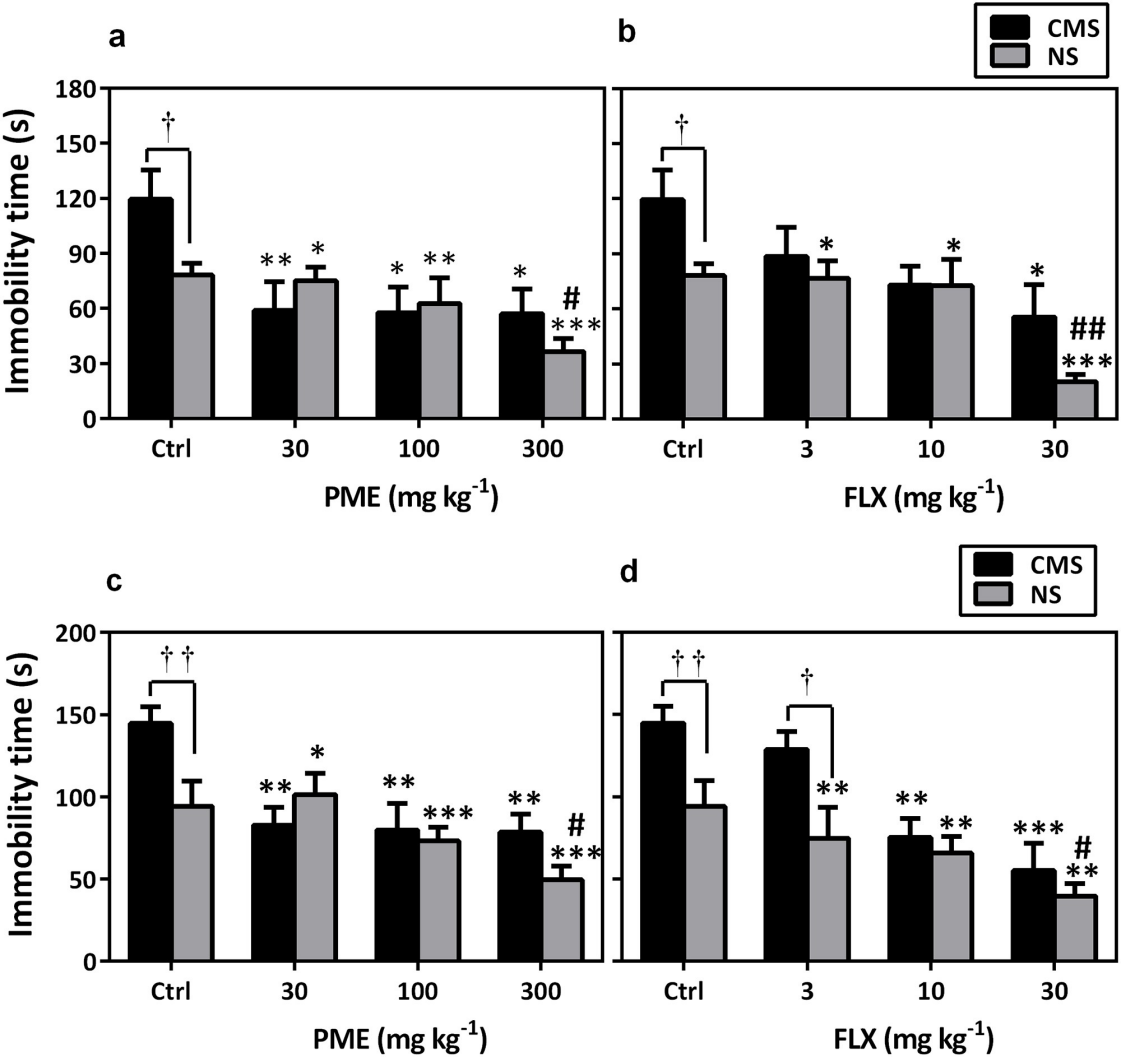
Effects of PME (30–300 mg kg^-1^) and FLX (3–30 mg kg^-1^) on immobility time in NS and CMS mice in the TST (a and b) and FST (c and d). Data are presented as mean±SEM (n = 8). Significantly different from CMS control: *P<0.05, **P<0.01, ***P<0.001 (one-way ANOVA followed by Newman-Keuls test). Significant difference when CMS and NS groups were compared: †*P*<0.05, ††*P*<0.01 (two-way repeated measures ANOVA followed by Bonferroni’s *post hoc* test). #*P*<0.05, ##*P*<0.01: compared to NS-control group. ctrl, control.

Mice with CMS-induced depression exhibited a significant increase in immobility duration as compared to the control group (*P*<0.05). However, treatment with PME for six weeks significantly reversed the increased immobility time (F_4,35_ = 3.872, *P* = 0.010; Fig 6a) induced by CMS. In the NS mice, PME significantly decreased immobility time as compared to the NS group with Newman-Keuls *post hoc* analysis revealing a statistical significance at 300 mg kg^-1^ (*P*<0.05). Immobility duration was also significantly decreased (F_4,35_ = 2.932, *P* = 0.0343; Fig 6b) by FLX in CMS mice when compared to the stressed-control group.

### Forced swim test

Fig 6c and 6d shows the effects of PME and FLX on the duration of immobility in mice in the FST. A significant increase in immobility time (*P*<0.01) was observed in mice subjected to CMS; however, this was not observed in the NS control mice. Furthermore, chronic treatment with PME (30, 100, or 300 mg kg^-1^; *p*.*o*.) significantly decreased immobility duration (F_4,35_ = 4.631, *P* = 0.0042) in all stressed mice. In addition, daily treatment with PME resulted in a significant decrease in immobility time in NS mice (*P*<0.05) at 300 mg kg^-1^ when compared to the NS control group using *post hoc* analysis. The reference drug FLX (3, 10, or 30 mg kg^-1^; *p*.*o*.) also significantly decreased immobility duration (F_4,35_ = 7.632, *P* = 0.0001) in CMS mice.

### Open field test

In Table 1, the Newman-Keuls *post hoc* analysis showed that exposure of mice to the CMS paradigm resulted in decreased central ambulation (*P*<0.001) and rearing (*P*<0.001) when compared to the NS group. However, administration of PME to mice subjected to CMS resulted in a significant reversal of the stress-induced behavioral alteration as observed by increases in central ambulation (F_4,35_ = 6.73, *P* = 0.0004) and rearing (F_4,35_ = 6.012, *P* = 0.0009).

**Table 1:** Effects of PME and FLX on the behavior of NS and CMS mice in the OFT.

Groups	Dose (mg kg^-1^)	Central Activity (CA)	Total Activity (TA)	CA/TA	Rearing Frequency
**NS**		25.25±3.30***	122.3±10.25***	0.20±0.01**	22.75±1.82***
**CMS**		8.38±1.51	74.0±5.32	0.12±0.02	7.00±1.13
PME (NS)	30	24.63±2.59***	115.4±6.04***	0.21±0.02**	19.75±2.22***
	100	25.50±1.75***	116.3±3.18***	0.22±0.02**	20.13±2.22***
	300	26.63±3.01***	119.4±4.42***	0.22±0.03**	22.88±1.89***
PME (CMS)	30	14.25±1.84*†	85.50±7.00	0.17±0.02	15.13±2.35*
	100	15.25±2.54*	87.50±13.71	0.19±0.04	19.00±4.21**
	300	18.13±2.29*	86.38±10.79	0.24±0.03*	17.88±1.06**
FLX (NS)	3	24.00±4.42***	112.50±11.28**	0.21±0.03**	18.88±2.72**
	10	24.88±2.01***	114.10±4.99**	0.22±0.02**	19.63±3.02**
	30	28.43±2.34***	129.00±10.33**	0.22±0.01**	23.57±2.82***
FLX (CMS)	3	12.63±2.12**††**	84.50±11.60	0.15±0.02	17.13±4.16*
	10	15.88±2.32*	90.38±4.76	0.17±0.02	17.88±2.40*
	30	18.88±2.62*	88.13±16.69	0.24±0.03**	17.00±3.30*

Key: NS, non-stressed; CMS, chronic mild stress; CA, central ambulation; TA, total activity

Data are expressed as group mean±SEM (n = 8). Significantly different from CMS group: *P<0.05, **P<0.01, ***P<0.001 (one-way ANOVA followed by Newman-Keuls test). Significant difference when CMS and NS groups were compared: †*P*<0.05, ††*P*<0.01 (two-way repeated measures ANOVA followed by Bonferroni’s *post hoc* test).

Daily administration of FLX resulted in higher central ambulation (F_4,35_ = 6.819, *P* = 0.0004) and rearing (F_4,35_ = 4.278, *P* = 0.0064) in CMS mice as compared to the stressed-control group.

Total activity (number of crossings), which is a measure of exploratory behavior in the OFT, was significantly decreased in the CMS mice (*P*<0.05; as compared to the NS-control group). After six weeks of treatment with PME, *post hoc* analysis of the data obtained showed no significant difference in total activity (*P*>0.05, compared to the CMS or NS control group). Chronic treatment with FLX also had no effect on total activity (*P*>0.05).

With regards to the ratio of central ambulation to total activity, this was significantly decreased in the CMS mice (*P*<0.01; as compared to the NS-control group). One-way ANOVA showed that the ratio of central ambulation to total activity was significantly increased in mice subjected to CMS after chronic administration of PME (F_4,35_ = 2.94, *P* = 0.0340) or FLX (F_4,35_ = 4.57, *P* = 0.0045).

### EPM and NSF tests

**Fig 7:**
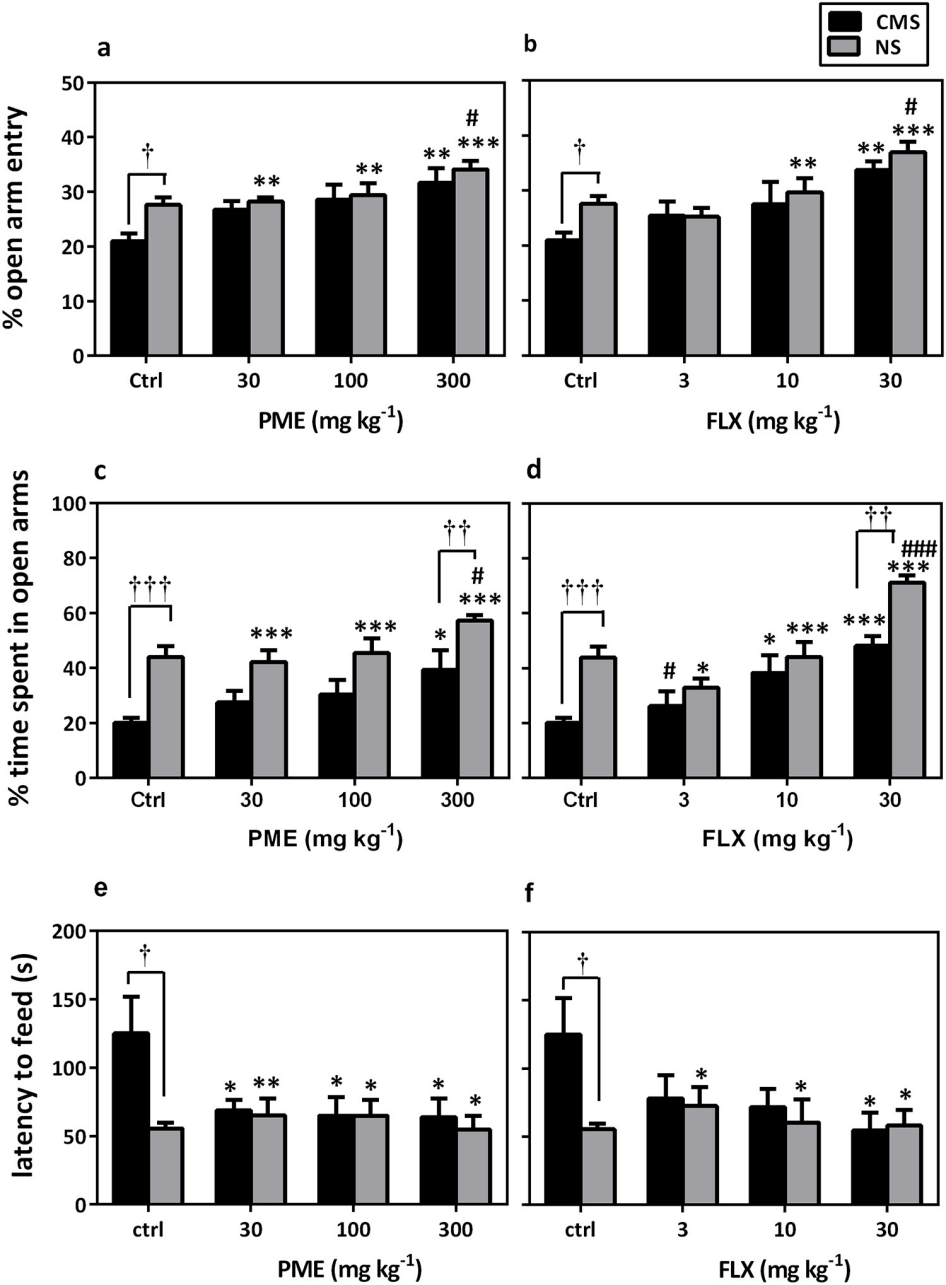
Effects of PME (30–300 mg kg^-1^) and FLX (3–30 mg kg^-1^) in NS and CMS-exposed mice on % open arm entries (a and b), % time spent in open arms (c and d) in the EPM test and latency to feed (e and f) in the NSF paradigm. Data are presented as mean±SEM (n = 8). Significantly different from CMS control: *P<0.05, **P<0.01, ***P<0.001 (one-way ANOVA followed by Newman-Keuls’ *post hoc* test). Significantly different from NS control: *#P*<0.05, ###*P*<0.001 (one-way ANOVA followed by Newman-Keuls’ *post hoc* test). Significantly different when CMS and NS groups were compared: †*P*<0.05, ††*P*<0.01, †††*P*<0.001 (two-way repeated measures ANOVA followed by Bonferroni’s *post hoc* test). ctrl, control.

In the EPM test, mice exposed to CMS were more anxious than the control mice, as can be observed by the decreases in % open arm entries and % time spent in open arms (Fig 7). This anxiety-like behavior was however reversed by daily treatment with PME, which resulted in significant increases in % open arm entries (F_4,35_ = 3.592, *P* = 0.014; Fig 7a) and % time spent in open arms (F_4,35_ = 9.967, *P* = 0.010; Fig 7c). Newman-Keuls *post hoc* analysis also revealed an anxiolytic-like activity among the PME-treated NS mice; this was evidenced by the statistically significant increases in % entries and % time spent in the open arms at 300 mg kg^-1^ (both at *P*<0.05 as compared to the NS group).

FLX induced similar results in the CMS mice as PME did [% open arm entries (F_4,35_ = 3.554, *P* = 0.015; Fig 7b) and % time spent in open arms (F_4,35_ = 9.497, *P* = 0.0003; Fig 7d)]. Chronic administration of FLX to NS mice also resulted in an anxiolytic-like effect as compared to the saline control group [% open arm entries (F_4,34_ = 9.923, *P*<0.0001) and % time spent in open arms (F_4,34_ = 24.31, *P*<0.0001)].

In the NSF test, exposure of mice to CMS significantly prolonged feeding latency (*P*<0.05 compared to NS control), whereas chronic treatment with either PME (F_4,35_ = 3.42, *P* = 0.018; Fig 7e) or FLX (F_4,35_ = 3.024, *P* = 0.030; Fig 7f) significantly decreased latency to feed in the novel arena.

### Morris water maze test

This test was performed to assess spatial learning and memory in the mice. As the number of trial days progressed, mice exposed to CMS showed increased escape latency (decreased learning behavior) as compared to the NS control group; this increase was statistically significant on the third day (*P*<0.05). However, PME and FLX attenuated the learning deficit induced by CMS. None of the mice showed significant changes in escape latency during the first day as compared to the CMS control group (*P*>0.05).

**Fig 8:**
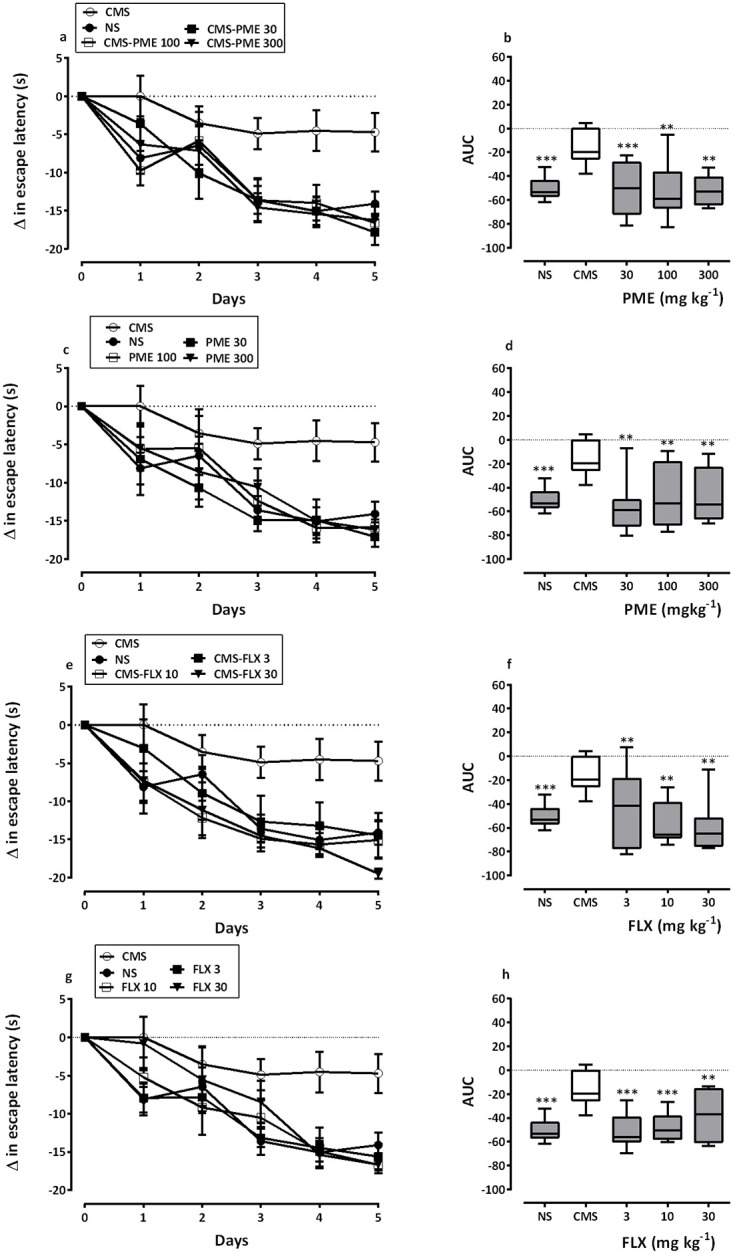
Performances of NS and CMS mice after chronic treatment with PME (30–300 mg kg^-1^) or FLX (3–30 mg kg^-1^) in the MWM test. Data are presented as time-course curves (a, c, e, and g) and as mean±SEM (n = 8) of AUCs (b, d, f, and h). Significantly different from CMS control group: **P<0.01, ***P<0.001 (one-way ANOVA followed by Newman-Keuls’ *post hoc* test). ctrl, control.

Change in escape latency decreased significantly in all drug-treated stressed mice following the training sessions, indicating that all mice showed some degree of learning [PME: F_4,209_ = 11.31, *P*<0.0001 (Fig 8a) and FLX: F_4,209_ = 12.72, *P*<0.0001 (Fig 8e); two-way ANOVA (*treatment × time*)]. Moreover, a *post hoc* analysis revealed significant difference in data from the third trial for all treated groups (*P*<0.05), which confirms good learning in mice exposed to various stressors. One-way ANOVA revealed a significant decrease in the change in escape latency following treatment with PME (F_4,35_ = 6.693, *P* = 0.0004; Fig 8b) and FLX (F_4,35_ = 5.898, *P* = 0.001; Fig 8f), which is indicative of an improvement in learning behavior.

**Fig 9:**
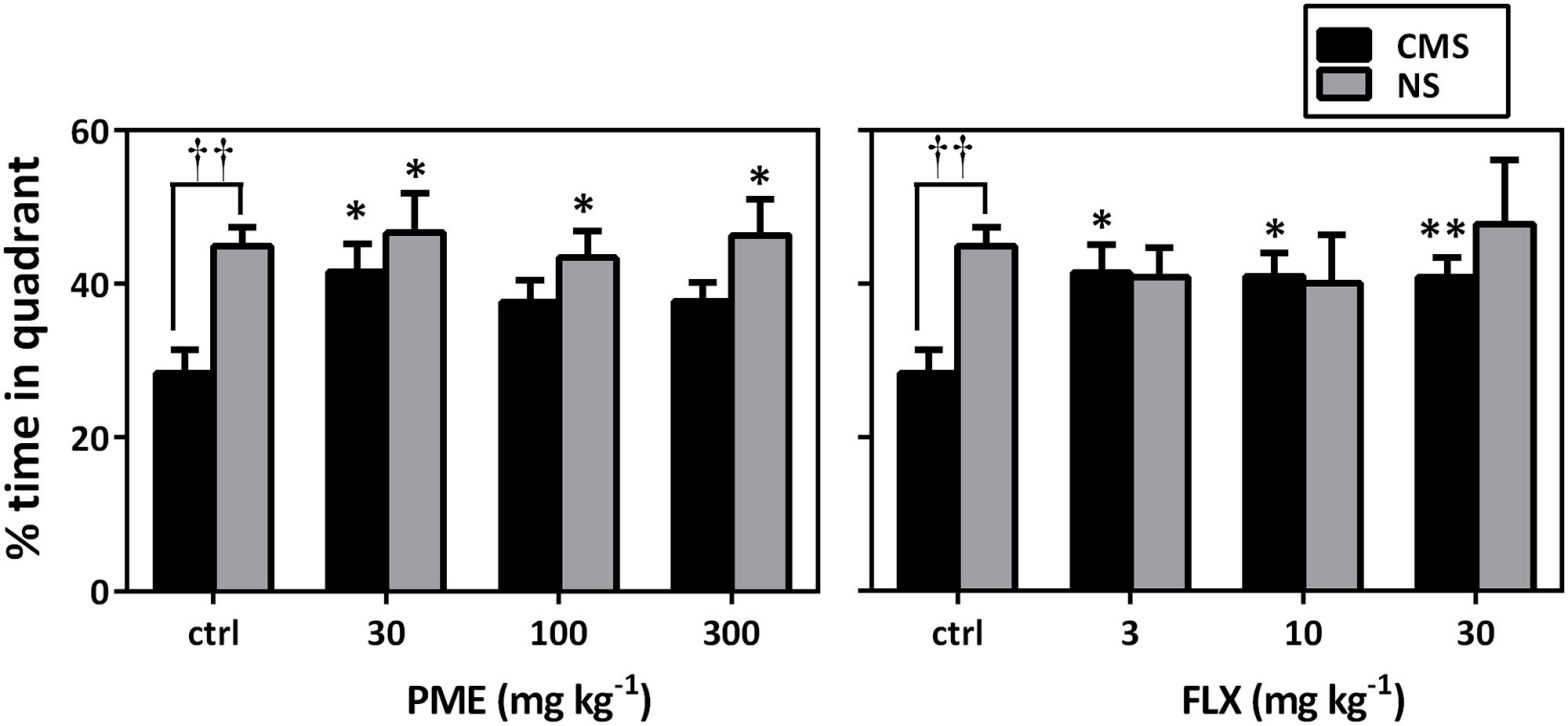
Effects of PME (30–300 mg kg^-1^) and FLX (3–30 mg kg^-1^) on the % time spent in target quadrant by NS and CMS mice in the probe trial test. Data are presented as mean±SEM (n = 8). Significantly different from CMS control: * *P*<0.05, ** *P*<0.01 (one-way ANOVA followed by Newman-Keuls test). Significant difference when CMS and NS groups were compared: ††*P*<0.01 (two-way repeated measures ANOVA followed by Bonferroni’s *post hoc* test). ctrl, control.

The spatial probe trial test was performed 24 h after the last training session to assess memory retention. CMS mice showed impaired cognitive performance; this was evidenced by a significant decrease in preference for the target quadrant, where the platform was previously placed during the training trials (*P*<0.01 compared to the NS control mice). Furthermore, PME (F_4,35_ = 4.421, *P* = 0.0054) and FLX (F_4,35_ = 4.496, *P* = 0.0049) significantly increased the percentage time spent in the target quadrant by the stressed mice, which is indicative of improved memory (Fig 9). However, chronic treatment with PME and FLX did not affect spatial learning or memory in the NS animals (*P*>0.05 compared to NS control mice).

### Elevated plus maze transfer latency

**Fig 10:**
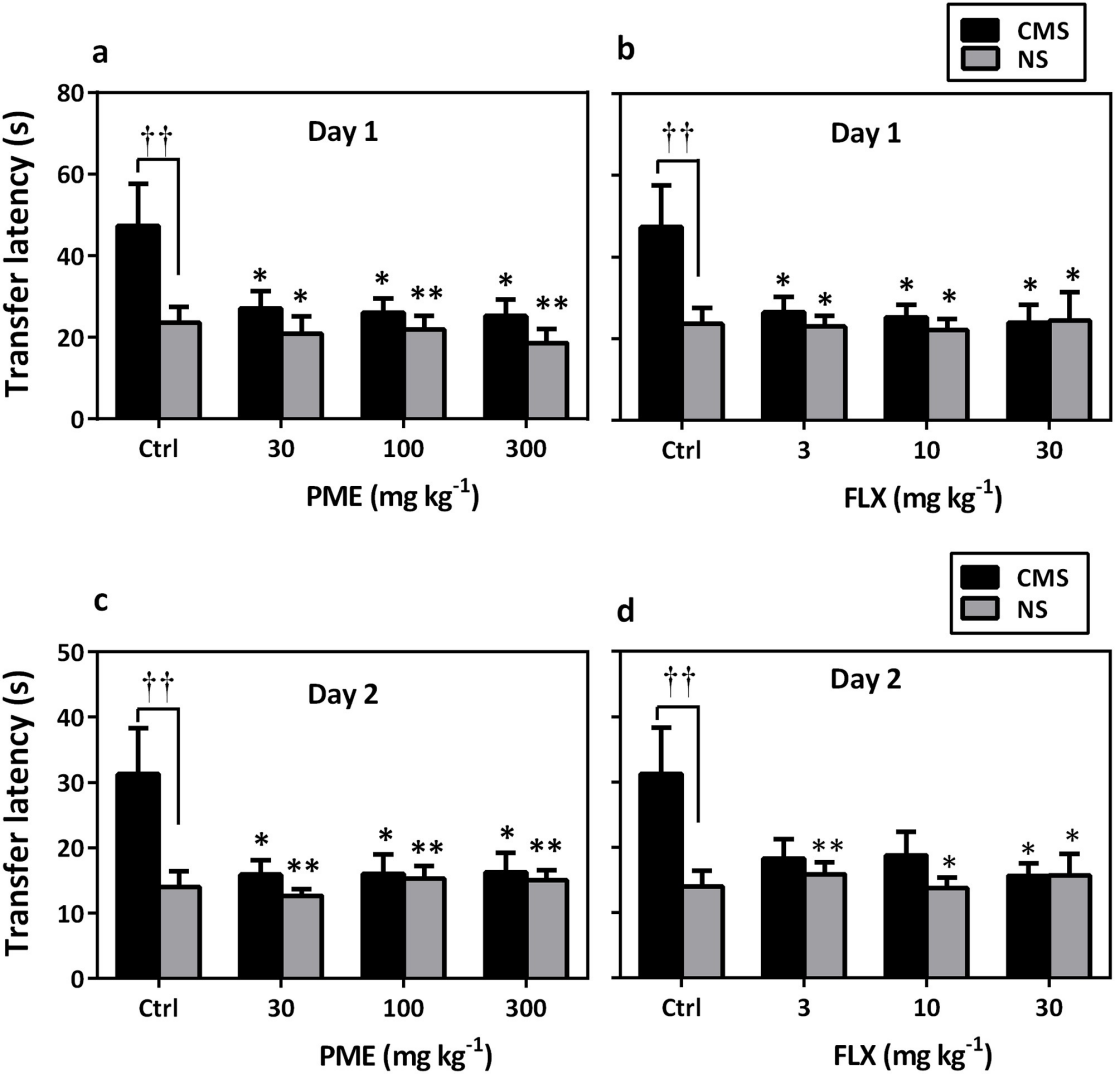
Performances of NS and CMS mice after chronic treatment with PME (30–300 mg kg^-1^) or FLX (3–30 mg kg^-1^) in the EPM transfer latency test. Data are presented as mean±SEM (n = 8). Significantly different from CMS control: * *P*<0.05, ** *P*<0.01 (one-way ANOVA followed by Newman-Keuls *post hoc* test). Significant difference when CMS and NS groups were compared: ††*P*<0.01 (two-way repeated measures ANOVA followed by Bonferroni’s *post hoc* test). ctrl, control.

Mice with CMS-induced depression showed significant increase in transfer latency on day 1 (learning) in the EPM test (*P*<0.05 compared to the NS control group). However, PME (*F*_4,35_ = 2.907, *P* = 0.035; Fig 10a) and FLX (*F*_4,35_ = 3.136, *P* = 0.026; Fig 10b) significantly decreased transfer latency in CMS mice. On day 2 (memory), the decreased transfer latency observed in CMS mice was significantly reversed by treatment with PME (*F*_4,35_ = 3.181, *P* = 0.0249; Fig 10c) and FLX (*F*_4,35_ = 2.836, *P* = 0.038; Fig 10d).

## Discussion

In this study, CMS consistently changed the behavioral profiles of the mice, including anhedonia (reduced SIT), depression (FST and TST), and anxiety (EPM, OFT, and NSF). In the MWM task, cognitive deficits were also noted. However, PME restored these behavioral modifications, indicating that the extract may be able to guard against the harmful effects of chronic depression.

For preclinical evaluation of antidepressants in animal models, chronic exposure to a variety of uncontrollable stimuli in an unpredictable way is well documented [13, 42]. Decreased sucrose consumption (sweet food) induced by the CMS model has been demonstrated to represent anhedonia (the lack of interest or pleasure) in animals, which is one of the key symptoms necessary for identifying a major depression in humans [43]. Interestingly, PME was able to reverse the anhedonic behavior induced by the CMS procedure. Anhedonia is a sign of depression; therefore, it stands to reason that PME exerted an antidepressant-like effect. Similar results were observed for FLX. The findings of this study support prior findings that repeated exposure to minor stresses over time resulted in a significant decrease in the consumption of 1% sucrose solution. This deficit can be efficiently corrected by long-term but not short-term usage of standard antidepressants [44].

According to numerous accounts, it normally takes 3–4 weeks of treatment to reverse CMS-induced anhedonia, which is very similar to the clinical time course of the effects of different antidepressants. [1, 45]. The action of antidepressants in CMS is similar to their clinical activity, with regards to their efficacy (full recovery at the end of treatment period) and specificity (lack of significant effects in NS animals) [12, 18]. Compared to the four weeks needed with FLX, anhedonic behavior in stressed mice receiving PME was reversed during the first two weeks of treatment. This suggests that PME will act more quickly than is often seen after continuous dosing of conventional antidepressants. Additionally, the effects of PME lasted during the entire duration of treatment. PME produced a comparable rapid-onset effects in our prior investigation using the repeated open-space swim paradigm, which involved the interaction of the extract with glycine/NMDA receptors, and this current study in CMS verifies these results [30]. Additionally, the results are consistent with other research demonstrating that glycine site NMDA antagonists had immediate antidepressant benefits in the CMS model of chronic depression [46, 47].

Furthermore, PME reversed CMS-induced immobility in the FST and TST, supporting the results of the weekly SIT tests, in which the extract greatly reduced anhedonia. To further confirm the antidepressant-like property of PME in CMS-induced depression, the coat state assessment and splash tests were conducted. Mice that are consistently stressed out typically have poor coat status, however antidepressant medications typically reverse this phenotype [48, 49]. The splash test on the other hand is both a direct measure of grooming and an indirect evaluation of SIT [9]. As anticipated, the CMS regimen used in this study caused coat state degradation and decreased grooming behavior in stressed mice during the splash test. However, PME treatment led to increased total grooming frequency and a better coat condition, which are signs of antidepressant-like activity. The findings of this investigation are consistent with previous studies in which antidepressants could prevent the impact of unexpected CMS on coat state or the splash test [32, 50].

Signs of anxiety are often present in both depressed patients and animal models for screening antidepressants. Furthermore, many antidepressant drugs have anxiolytic properties [51, 52] and are now recommended as first-line treatment for several anxiety disorders. Several studies have shown that exposure of mice to the CMS paradigm induces anxiety [36, 53]; therefore, the EPM, OFT, and NSF paradigms were performed to assess the impact of PME on CMS-induced anxiety. The OFT is a conventional approach/avoidance paradigm in which anxiety and exploration are simultaneously evoked by a novel setting [54]. An increase in activity or time spent in the center of the open field indicates reduction in anxiety and/or increase in exploration [55]. In this experiment, CMS mice repeatedly exposed to stressors showed reduced activity, central entries, and rearing, which are signs of increased anxiety and diminished exploration, which is consistent with other findings [56, 57]. However, PME greatly increased central ambulation and rearing to counteract the altered open field behavior, most likely as a result of its anxiolytic impact [58]. Furthermore, PME administration over an extended period of time restored the CMS-induced reduction in locomotor activity. As compared to the extract, the common antidepressant FLX had similar effects. This discovery is in line with other findings, which strongly imply that mice treated chronically with FLX have reduced anxiety and/or increased exploration [55].

The EPM test is a popular paradigm that offers an impartial assessment of rodents’ anxiety-like behavior (% of entry or time spent in the open arms) [59, 60]. Increased anxiety-like behavior in this test has been observed after CMS induction [61, 62]. The open arms of the EPM are naturally avoided by animals displaying anxiety-like behavior, and anxiolytic drugs often increase open arm exploration [63, 64]. According to the current research, animals exposed to CMS exhibit a marked reduction in the percentage of entries and duration spent in the EPM’s open arms, which is suggestive of an anxious state. Chronic administration of PME and FLX, however, markedly reduced this anxiogenic behavior, indicating an anxiolytic-like effect. In a prior investigation, the anxiolytic-like effects of PME in the EPM were confirmed [58].

The anxiolytic effect of PME was further evaluated in stressed and NS mice using the NSF test.

This test evaluates hyponeophagia, which is the inhibition of feeding brought on by novelty exposure. In a novel environment, anxiogenic treatments increase latency and decrease consumption while anxiolytics generally decrease latency and increase consumption [65]. In the current investigation, CMS increased the anxiogenic effects on the mice, but PME decreased the animals’ feeding latency (anxiolytic effect). According to earlier research, which shows that long-term use of serotonin-based antidepressants is necessary for the production of anxiolytic effects, reversal of the hyponeophagia caused by CMS can be accomplished by using PME [55, 66]. We have demonstrated previously that PME elicits its antidepressant-like effects via serotonergic mechanisms [29], hence, it is not surprising that it shows significant reduction in anxiety-related behaviors when tested in the three different approaches.

In humans, cognitive impairment and depression frequently coexist, and this association between the two conditions is notable. Dementia and depression may interact in a number of ways; for example, depression may be induced by cognitive decline, and dementia may also be a symptom of depression [67, 68]. Therefore, in this chronic model of depression, the effects of PME on cognitive performance in the MWM task were evaluated. Animals must employ additional maze cues to remember the position of a concealed platform in a pool of water in order to complete the hippocampal-dependent spatial learning and memory challenge known as MWM [69, 70]. Memory is time spent in the area of the platform during a test session in which the platform has been removed, whereas learning is assessed as a decreased latency to discover the hidden platform across sessions [71].

According to the results of this study, chronically subjecting mice to a variety of unpredictable stressors results in an increase in transfer latency and a decrease in quadrant dwell time during the MWM task, providing clear evidence that CMS significantly degrades hippocampal-dependent spatial learning and memory performance [1, 68]. The escape latencies in the drug-treated groups, however, steadily decreased as the number of training days increased, and there was a noticeable improvement over the last three days. Additionally, mouse performance in the probing trial, in which quadrant stay duration was considerably longer in mice treated with the extract, served as a primary indicator of the permanence of spatial memory. The results of this study thus show that repeated PME treatment may be therapeutically efficient in treating memory impairment caused by chronic stress.

We additionally measured weight gain or loss since it is a common symptom of depression [72, 73]. The current study demonstrated that CMS can significantly reduce mice’s body weight. Chronic stress has been shown in the past to change the rate of weight gain, especially in male rats [74, 75]. This reduction in weight gain is a reliable index of the stress experience [56, 76]. However, prolonged PME treatment increased weight gain in the stressed mice without having an impact on the control animals.

## Conclusion

The present study provides evidence that *P*. *microcarpa* leaf extract improves anhedonia, anxiety, and memory impairment induced by chronic exposure to mild stressors. Moreover, unlike FLX, it exhibits a rapid and sustained antidepressant-like effect.

## Supporting information

S1 Appendix Peak table for IR spectra of the hydroethanolic leaf extract of *P*. *microcarpa* (PME). (TIF)

S2 Appendix Baseline sucrose intake for mice (group 1). (PDF)

S3 Appendix Baseline sucrose intake for mice (group 2). (PDF)

S4 Appendix Sucrose intake for CMS mice after 3 weeks of baseline. (PDF)

S5 Appendix Sucrose intake for non-stressed after 3 weeks of baseline. (PDF)
