# Supplementary figures and images for: Fast-onset effects of Pseudospondias microcarpa (A. Rich) Engl. (Anacardiaceae) hydroethanolic leaf extract on behavioral alterations induced by chronic mild stress in mice
Source: PLoS One. 2023 Feb 2;18(2):e0278231. doi: 10.1371/journal.pone.0278231 (PMC9894402; doi:10.1371/journal.pone.0278231)

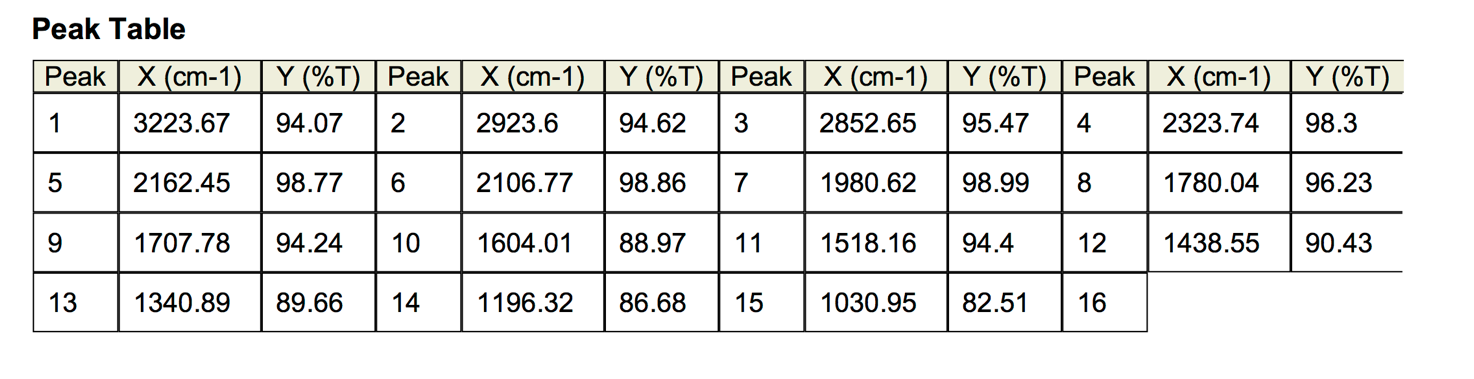

Supplement: S1 Appendix — (TIF) [file pone.0278231.s001.tif]
