# Supplementary material for: Fast-onset effects of Pseudospondias microcarpa (A. Rich) Engl. (Anacardiaceae) hydroethanolic leaf extract on behavioral alterations induced by chronic mild stress in mice
Source: PLoS One. 2023 Feb 2;18(2):e0278231. doi: 10.1371/journal.pone.0278231 (PMC9894402; doi:10.1371/journal.pone.0278231)
Supplement: S3 Appendix — (PDF) [file pone.0278231.s003.pdf]

									Baseline sucrose intake (group 2)						
		DAY 1			DAY 2			DAY 3			DAY 4			DAY 5	
	INITIAL	FINAL	Sucrose intake	INITIAL	FINAL	Sucrose intake	INITIAL	FINAL	Sucrose intake	INITIAL	FINAL	Sucrose intake	INITIAL	FINAL	Sucrose intake
A 1	147.11	144.88	2.23	152.39	150.32	2.07	129.35	125.68	3.67	144.98	142.94	2.04	146.16	144.31	1.85
2	146.7	142.58	4.12	151.65	148.09	3.56	131.97	127.53	4.44	147.71	144.3	3.41	145.8	142.73	3.07
3	131.48	127.28	4.2	140.75	138.18	2.57	130.63	127.05	3.58	146.33	143.53	2.8	141.02	137.79	3.23
4	146.84	144.36	2.48	141.26	138.69	2.57	134.63	130.49	4.14	143.23	140.59	2.64	149.18	146.31	2.87
5	143.08	139.82	3.26	138.78	136.43	2.35	130.48	127.07	3.41	156.27	152.71	3.56	145.3	142.18	3.12
6	164.28	160.47	3.81	166.81	164.38	2.43	147.79	144.46	3.33	163.18	160.46	2.72	160.39	157.32	3.07
7	172.25	170.05	2.2	167.2	165.09	2.11	149.69	145.82	3.87	156.96	154.32	2.64	174.94	172.41	2.53
8	140.04	137.07	2.97	134.73	132.11	2.62	150.9	147.92	2.98	141.85	139.36	2.49	153.45	151.27	2.18
B 1	140.71	136.18	4.53	133.49	131.55	1.94	144.59	141.04	3.55	144.63	142.14	2.49	134.98	132.8	2.18
2	129.03	126.45	2.58	139.35	137.34	2.01	142.89	138.7	4.19	136.09	132.76	3.33	161.83	158.76	3.07
3	141.6	138.31	3.29	141.1	139.1	2	145.17	141.77	3.4	144.57	142.15	2.42	149.32	147.13	2.19
4	140.31	138.46	1.85	136.32	134.34	1.98	134.91	130.45	4.46	148.46	145.35	3.11	155.53	151.96	3.57
5	141.47	138.6	2.87	140.29	138.11	2.18	141.22	136.63	4.59	150.16	148.04	2.12	154.14	151.65	2.49
6	161.2	159.17	2.03	160.73	158.85	1.88	166.45	163.48	2.97	167.59	165.37	2.22	159.85	157.7	2.15
7	162.71	159.49	3.22	147.48	145.24	2.24	161.41	157.6	3.81	161.6	158.7	2.9	155.86	153.06	2.8
8	149.05	146.73	2.32	144.65	142.47	2.18	140.82	137.5	3.32	135.5	133.11	2.39	143.06	140.7	2.36
C 1	140.06	138.12	1.94	139.59	137.69	1.9	139.24	135.88	3.36	133.69	130.81	2.88	147.76	145.12	2.64
2	139.91	136.44	3.47	134.7	132.73	1.97	150.98	147.83	3.15	142.75	140.52	2.23	142.3	140.23	2.07
3	141.71	139.83	1.88	136.77	135.04	1.73	149.5	146.71	2.79	144.42	142.02	2.4	143.06	140.48	2.58
4	172.56	170.43	2.13	164.78	162.19	2.59	174.34	170.81	3.53	172.9	170.35	2.55	183.3	180.98	2.32
5	130.41	127	3.41	135.69	133.07	2.62	149.75	146.25	3.5	147.57	144.27	3.3	135.5	132.65	2.85
6	171.1	169.84	1.26	161.94	160.13	1.81	169.32	165.98	3.34	157.01	155.13	1.88	163.47	161.28	2.19
7	153.18	150.78	2.4	147.39	144.97	2.42	157.66	154.57	3.09	145.59	142.9	2.69	148.78	145.09	3.69
8	168.57	166.92	1.65	163.67	162.41	1.26	180.82	178.74	2.08	167.89	166.68	1.21	157.08	154.85	2.23
D 1	141.28	138.65	2.63	139.02	137.38	1.64	144.16	140.22	3.94	146.61	143.96	2.65	154.71	152.26	2.45
2	172.69	171.02	1.67	161	158.61	2.39	161.08	157.64	3.44	156	153.34	2.66	154.45	151.54	2.91
3	155.03	152.58	2.45	147.62	143.93	3.69	154.94	151.22	3.72	141.6	138.63	2.97	151.21	148.78	2.43
4	137	134.31	2.69	141.87	139.68	2.19	150.62	146.86	3.76	140.19	137.94	2.25	143.63	141.09	2.54
5	140.55	138.61	1.94	144.13	142.16	1.97	157.59	153.92	3.67	144.35	141.92	2.43	153.82	150.88	2.94
6	146.61	143.86	2.75	135.94	133.08	2.86	152.74	148.44	4.3	153.98	150.55	3.43	145.97	142.82	3.15
7	145.66	143.8	1.86	137.66	134.85	2.81	153.9	150.68	3.22	140.48	137.69	2.79	147.39	144.69	2.7
8	149.16	146.64	2.52	134.97	131.86	3.11	145.3	142.48	2.82	139.76	137.5	2.26	146.33	144.38	1.95
E 1	164.36	162.8	1.56	153.5	151.35	2.15	166.67	163.53	3.14	158.71	156.79	1.92	148.12	145.75	2.37
2	141.87	140.2	1.67	135.36	132.97	2.39	154.29	151.02	3.27	141.59	138.87	2.72	146.53	143	3.53
3	175.84	174.28	1.56	172.03	169.57	2.46	183.35	181.41	1.94	165.73	164.53	1.2	170.56	168.78	1.78
4	154	152.32	1.68	141.28	139.2	2.08	139.24	136.03	3.21	130.48	127.45	3.03	148.56	145.08	3.48
5	158.98	157.34	1.64	140.31	137.28	3.03	150.87	146.15	4.72	132.41	128.74	3.67	139.9	136.8	3.1
6	159.57	157.78	1.79	140.61	139.66	0.95	149.57	145.3	4.27	130.09	127.32	2.77	145.32	142.18	3.14
7	142.22	139.03	3.19	137.18	134.9	2.28	154.48	151.24	3.24	150.86	148.63	2.23	149.83	147.15	2.68
8	156.58	154.33	2.25	139.3	136.71	2.59	153.32	149.87	3.45	146.05	144.05	2	158.62	156.2	2.42
F 1	149.38	147.36	2.02	143.68	141.6	2.08	156	153.63	2.37	149.45	147.82	1.63	162.47	160.43	2.04
2	161.08	159.14	1.94	135.22	133.34	1.88	160.56	157.58	2.98	157.24	155.54	1.7	154.4	152.86	1.54
3	189.59	187.95	1.64	162.73	159.94	2.79	182.59	178.76	3.83	177.06	173.9	3.16	170.55	167.77	2.78
4	184.82	182.65	2.17	165.42	163.57	1.85	173.83	171.26	2.57	170.03	168.33	1.7	168.69	166.94	1.75
5	175.7	172.46	3.24	169.5	167.04	2.46	170.22	165.79	4.43	175.85	172.89	2.96	162.61	159.51	3.1
6	154.99	153.21	1.78	134.93	133.13	1.8	149.11	146.03	3.08	148.31	146.63	1.68	149.42	147.5	1.92
7	162.15	160.01	2.14	140.88	138.71	2.17	163.68	160.18	3.5	153.25	149.86	3.39	153.58	151.03	2.55
8	157.1	155.32	1.78	141.85	139.48	2.37	166.83	164.76	2.07	152.86	149.99	2.87	156.08	153.94	2.14
G 1	163.29	161.43	1.86	140.78	137.8	2.98	149.12	145.15	3.97	152.46	149.85	2.61	164.28	162.06	2.22
2	176.68	175.33	1.35	136.5	133	3.5	159.71	154.68	5.03	150.57	146.31	4.26	149.57	145.9	3.67
3	205.79	203.76	2.03	171.01	168.23	2.78	189.51	185.55	3.96	180.69	177.84	2.85	189.32	186.69	2.63
4	165.23	162.02	3.21	138.84	135.62	3.22	158.46	154.31	4.15	157.52	154.75	2.77	165.45	163.43	2.02
5	167.02	164.52	2.5	142.63	139.74	2.89	163.9	160.53	3.37	157.24	154.99	2.25	167.11	163.2	3.91
6	173.74	172.28	1.46	137.95	136.33	1.62	165.91	161.49	4.42	162.85	160.65	2.2	160.31	157.76	2.55
7	166.11	164.42	1.69	136.85	135.39	1.46	153.54	150.32	3.22	156.65	153.72	2.93	153.31	150.91	2.4
8	162.25	160.1	2.15	141.3	138.34	2.96	168.8	165.5	3.3	175.32	172.31	3.01	166.3	163.1	3.2
