# Supplementary material for: Fast-onset effects of Pseudospondias microcarpa (A. Rich) Engl. (Anacardiaceae) hydroethanolic leaf extract on behavioral alterations induced by chronic mild stress in mice
Source: PLoS One. 2023 Feb 2;18(2):e0278231. doi: 10.1371/journal.pone.0278231 (PMC9894402; doi:10.1371/journal.pone.0278231)
Supplement: S4 Appendix — (PDF) [file pone.0278231.s004.pdf]

						Sucrose intake for CMS group							
	STRESSED		WK 1				WK 2				WK 3		
		INITIAL	FINAL	Sucrose intake		INITIAL	FINAL	Sucrose intake		INITIAL	FINAL	Sucrose intake	
SA 1		80.95	78.18	2.77		104.27	102.83	1.44		75.79	73.92	1.87	
2		76.54	74.11	2.43		78.73	77.22	1.51		80.25	78.91	1.34	
3		80.86	78.05	2.81		87.41	85.86	1.55		82.51	80.08	2.43	
4		77.37	76.04	1.33		90.72	88.68	2.04		85.77	84.77	1	
5		77.46	76.04	1.42		93.2	91.55	1.65		87.7	85.66	2.04	
6		80.62	79.04	1.58		92.39	90.26	2.13		80.22	79.04	1.18	
7		78.08	75.52	2.56		91.83	89.75	2.08		80.12	78.23	1.89	
8		80.06	78	2.06		101.66	100	1.66		77.27	75.5	1.77	
SB 1		83.53	81.55	1.98		96.45	94.2	2.25		79.74	78.59	1.15	
2		73.23	71.39	1.84		89.85	88.12	1.73		77.5	75.6	1.9	
3		82	79.82	2.18		77.73	76.02	1.71		78.63	76.44	2.19	
4		84.88	82.87	2.01		90.7	88.49	2.21		80.4	78.88	1.52	
5		75.3	73.63	1.67		94.06	92.9	1.16		81.05	79.57	1.48	
6		89.72	87.86	1.86		79.61	78.53	1.08		75.82	73.84	1.98	
7		80.96	78.88	2.08		89.23	86.39	2.84		88	86.04	1.96	
8		83.26	80.81	2.45		93.83	92.79	1.04		93.84	92.02	1.82	
SC 1		76.1	74.84	1.26		72.43	70.47	1.96		79.3	77.08	2.22	
2		81.6	79.62	1.98		84.56	83.42	1.14		78.06	75.66	2.4	
3		77.1	75.35	1.75		96.63	94.58	2.05		75.95	73.52	2.43	
4		70.07	67.91	2.16		78.5	76.8	1.7		87.41	85.09	2.32	
5		88.95	86.66	2.29		85.18	84.04	1.14		73.37	70.97	2.4	
6		84.99	82.9	2.09		101.6	100.26	1.34		85.41	83.08	2.33	
7		73.72	72.32	1.4		99.54	98.34	1.2		84.34	82.13	2.21	
8		82.44	80.23	2.21		94.87	92.98	1.89		93.4	91.4	2	
SD 1		75.53	73.69	1.84		88.56	87.14	1.42		82.96	80.87	2.09	
2		68.74	67.05	1.69		87.76	85.88	1.88		87.12	84.75	2.37	
3		73.37	71.18	2.19		91.16	89.54	1.62		88.79	86.6	2.19	
4		62.89	60.81	2.08		91.08	90.15	0.93		74.17	72.19	1.98	
5		72.79	71.09	1.7		86.4	85.26	1.14		83.88	81.34	2.54	
6		81.33	79.12	2.21		105.5	104.38	1.12		90.09	88.06	2.03	
7		72.95	71.06	1.89		99	97.89	1.11		79.44	77.5	1.94	
8		66.92	64.52	2.4		99.84	98.86	0.98		74.74	73.85	0.89	
SE 1		78.21	75.61	2.6		88.6	86.79	1.81		80.06	79.18	0.88	
2		70.21	67.98	2.23		96.79	94.82	1.97		77.26	75.95	1.31	
3		71.5	70.09	1.41		98.47	97.55	0.92		79.6	76.86	2.74	
4		72.7	72.47	0.23		93.59	91.87	1.72		87.76	85.71	2.05	
5		69.86	67.5	2.36		90.84	88.21	2.63		73.94	71.95	1.99	
6		66.94	64.89	2.05		80	78.53	1.47		83.6	81.25	2.35	
7		73.62	71.83	1.79		86.21	84.96	1.25		73.04	71.62	1.42	
8		76.1	74.62	1.48		94.05	92.56	1.49		83.41	81.52	1.89	
SF1		68.21	66.08	2.13		83.64	80.64	3		72.6	69.93	2.67	
2		74.24	72.5	1.74		86.01	84.53	1.48		72.65	70.74	1.91	
3		68.12	66.61	1.51		80.98	79.6	1.38		81.79	79.99	1.8	
4		60.7	58.78	1.92		75.33	73.8	1.53		82.39	79.76	2.63	
5		71.84	70.49	1.35		77.87	75.91	1.96		75.87	73.88	1.99	
6		67.58	65.73	1.85		78.69	78.19	0.5		74.63	72.42	2.21	
7		60.77	59.16	1.61		76.96	75.89	1.07		69.84	68.06	1.78	
8		65.2	63.37	1.83		84.37	83.27	1.1		75.78	74.08	1.7	
SG 1		79.23	77.8	1.43		75.66	73.87	1.79		73.9	72.56	1.34	
2		82.06	80.1	1.96		73.95	71.19	2.76		75.84	74.27	1.57	
3		72.28	70.18	2.1		74.92	73	1.92		73.52	71.93	1.59	
4		66.17	64.85	1.32		73.65	72	1.65		77.12	75.54	1.58	
5		78.17	76.58	1.59		78.01	75.81	2.2		77.23	74.56	2.67	
6		71.43	69.99	1.44		94.62	93.29	1.33		70.33	68.43	1.9	
7		71.74	69.26	2.48		74.95	73.14	1.81		68.96	67.31	1.65	
8		76.48	75	1.48		85.15	84.01	1.14		76.46	75.02	1.44	
