# Supplementary material for: Fast-onset effects of Pseudospondias microcarpa (A. Rich) Engl. (Anacardiaceae) hydroethanolic leaf extract on behavioral alterations induced by chronic mild stress in mice
Source: PLoS One. 2023 Feb 2;18(2):e0278231. doi: 10.1371/journal.pone.0278231 (PMC9894402; doi:10.1371/journal.pone.0278231)
Supplement: S5 Appendix — (PDF) [file pone.0278231.s005.pdf]

						sucrose intake for naïve control							
	CONTROL		WK 1				WK 2				WK 3		
		INITIAL	FINAL	Sucrose intake		INITIAL	FINAL	Sucrose intake		INITIAL	FINAL	Sucrose intake	
A 1		70.7	67.92	2.78		85.93	83.33	2.6		76.32	73.14	3.18	
2		76.14	73.47	2.67		83.15	80.22	2.93		83.21	80.33	2.88	
3		80.56	78.36	2.2		81.27	79.68	1.59		71.66	68.51	3.15	
4		70.6	67.97	2.63		80.63	78.47	2.16		74.83	71.58	3.25	
5		74.9	71.94	2.96		76.88	72.65	4.23		75	71.84	3.16	
6		69.9	68.38	1.52		89.12	87.26	1.86		77.47	75.01	2.46	
7		73.62	71.83	1.79		90.86	89.07	1.79		80.17	77.67	2.5	
8		76.88	74.7	2.18		77.44	75.39	2.05		76.64	74.5	2.14	
B 1		78.95	76.06	2.89		73.54	71.1	2.44		70.04	66.63	3.41	
2		73.47	71.67	1.8		86.42	84.57	1.85		68.15	66.05	2.1	
3		70.55	68.28	2.27		77.09	75.34	1.75		80.81	77.81	3	
4		72.63	69.73	2.9		77.58	74.96	2.62		77.45	74.61	2.84	
5		80.17	76.4	3.77		75.65	73.48	2.17		85.35	81.22	4.13	
6		73.27	70.49	2.78		86.74	84.6	2.14		87.7	84.93	2.77	
7		77.32	74.54	2.78		91.86	89.77	2.09		76.7	73.96	2.74	
8		77.45	74.95	2.5		90.82	88.95	1.87		70.15	67.04	3.11	
C 1		74.52	72.49	2.03		91.47	89.42	2.05		78.93	76.69	2.24	
2		74.49	71.96	2.53		79.43	77.17	2.26		79.2	76.6	2.6	
3		82.92	80.8	2.12		79.11	77.25	1.86		70.23	67.03	3.2	
4		83.81	81.45	2.36		86.64	83.98	2.66		88.25	84.73	3.52	
5		77.74	75.34	2.4		88.83	86.87	1.96		84.27	80.75	3.52	
6		83.95	81.93	2.02		86.35	84.07	2.28		82.75	80.06	2.69	
7		93.41	91.09	2.32		83.95	81.44	2.51		80.19	76.99	3.2	
8		90.93	88.93	2		88	86.57	1.43		71.35	69.04	2.31	
D 1		74.57	71.98	2.59		86.6	84.18	2.42		85.11	81.77	3.34	
2		84.03	81.23	2.8		97.8	95.42	2.38		83.44	79.94	3.5	
3		76.97	74.11	2.86		90.53	88.64	1.89		81.31	78.38	2.93	
4		77.95	75.74	2.21		81.27	79.1	2.17		79.1	75.16	3.94	
5		83.97	81.99	1.98		100.49	98.44	2.05		78.16	75.72	2.44	
6		87.83	85.25	2.58		93.75	91.66	2.09		87.16	84.26	2.9	
7		71.1	69.1	2		83.14	80.72	2.42		87.15	85.09	2.06	
8		80.97	78.21	2.76		80.88	78.34	2.54		86.63	84.33	2.3	
E 1		75.52	73.32	2.2		83.73	81.3	2.43		82.34	80.05	2.29	
2		83.89	81.05	2.84		84.33	80.79	3.54		71.5	68.33	3.17	
3		79.19	77.46	1.73		87.22	86.19	1.03		81.31	79.06	2.25	
4		90.69	88.66	2.03		80.41	79.12	1.29		92.88	90.87	2.01	
5		74.32	72.75	1.57		102.08	100.01	2.07		90.65	87.64	3.01	
6		94.22	91.32	2.9		101.08	98.34	2.74		79.08	75.61	3.47	
7		100.93	98.8	2.13		87.96	84.42	3.54		89.02	85.47	3.55	
8		88.62	86.41	2.21		93.96	91.65	2.31		79.2	77.13	2.07	
F1		89.94	87.74	2.2		93.24	91	2.24		85.93	83.05	2.88	
2		84.37	82.78	1.59		84.28	82.13	2.15		86.2	83.98	2.22	
3		77.05	73.96	3.09		94.37	91.54	2.83		93.64	90.2	3.44	
4		96.97	94.36	2.61		85.82	83.98	1.84		73.96	71.7	2.26	
5		92.77	90.43	2.34		90.58	86.79	3.79		80.53	78.07	2.46	
6		98.81	96.17	2.64		105.63	103.36	2.27		85.25	82.77	2.48	
7		80.79	78.05	2.74		90.87	88.2	2.67		79.24	76.73	2.51	
8		99.6	97.56	2.04		91.2	89.16	2.04		81.7	79.36	2.34	
G 1		87.86	85.09	2.77		116.72	113.91	2.81		89.66	87.12	2.54	
2		92.61	89.96	2.65		119.8	117.4	2.4		92.71	89.87	2.84	
3		91.68	89.17	2.51		102.93	100.19	2.74		83.73	80.71	3.02	
4		91.7	88.98	2.72		103.52	101.06	2.46		91.71	89.6	2.11	
5		93.27	90.52	2.75		100.93	98.29	2.64		85.64	83.59	2.05	
6		90.7	88.03	2.67		98.21	96.18	2.03		101.7	98.8	2.9	
7		97.6	95.22	2.38		96.66	93.92	2.74		106.51	103.84	2.67	
8		92.52	90.12	2.4		100.36	97.52	2.84		95.61	93.2	2.41	
