# Supplementary material for: Fast-onset effects of Pseudospondias microcarpa (A. Rich) Engl. (Anacardiaceae) hydroethanolic leaf extract on behavioral alterations induced by chronic mild stress in mice
Source: PLoS One. 2023 Feb 2;18(2):e0278231. doi: 10.1371/journal.pone.0278231 (PMC9894402; doi:10.1371/journal.pone.0278231)
Supplement: S2 Appendix — (PDF) [file pone.0278231.s002.pdf]

Baseline sucrose intake (group 1)

	DAY 1			DAY 2			DAY 3			DAY 4			DAY 5		
	INITIAL	FINAL	Sucrose intake	INITIAL	FINAL	Sucrose intake	INITIAL	FINAL	Sucrose intake	INITIAL	FINAL	Sucrose intake	INITIAL	FINAL	Sucrose intake
SA 1	163.9	160.84	3.06	140.75	138.18	2.57	148.44	145.22	3.22	168.1	164.31	3.79	162.05	160.03	2.02
2	152.01	148.38	3.63	137.54	134.41	3.13	170.98	167.75	3.23	153.28	151.16	2.12	157.07	155	2.07
3	154.51	150.84	3.67	144.88	142.44	2.44	142.05	139.65	2.4	162.01	159.08	2.93	160.95	158.11	2.84
4	156.86	153.79	3.07	148.2	145.6	2.6	146.37	143.32	3.05	150.33	146.39	3.94	153.38	150.33	3.05
5	163.61	160.31	3.3	147.03	143.72	3.31	144.73	141.3	3.43	155.8	152.12	3.68	167.55	164.33	3.22
6	182.25	180.18	2.07	164.88	161.8	3.08	141.7	138.61	3.09	154.3	152.82	1.48	177.86	174.66	3.2
7	176.82	173.58	3.24	171.6	168.26	3.34	157.13	153.43	3.7	175.72	173.22	2.5	177.01	174.08	2.93
8	155.42	152.55	2.87	139.35	135.8	3.55	172.11	169.09	3.02	149.8	147.31	2.49	149.67	146.18	3.49
SB 1	147.82	145.33	2.49	147.59	144.52	3.07	148.18	144.91	3.27	163.1	161.87	1.23	144.32	141.5	2.82
2	162.18	158.06	4.12	156.48	152.93	3.55	151.05	147.69	3.36	156.55	153.31	3.24	181.9	177.96	3.94
3	141.94	139	2.94	139.33	136.07	3.26	146.01	142.2	3.81	150.54	148.55	1.99	157.75	154.56	3.19
4	138.39	135.22	3.17	143.16	139.98	3.18	136.78	134.73	2.05	142.44	139.67	2.77	156.08	152.59	3.49
5	143.93	141.17	2.76	149.49	146.7	2.79	147	143.64	3.36	160.43	157.85	2.58	157.76	154.8	2.96
6	155.14	152.97	2.17	142.01	139.16	2.85	171.01	167.02	3.99	165.2	163.02	2.18	157.1	154.7	2.4
7	151.1	148.09	3.01	152.88	149.51	3.37	165.06	162.84	2.22	162.72	159.91	2.81	149.17	144.97	4.2
8	153.12	150.8	2.32	158.56	155.68	2.88	140.69	136.97	3.72	170.47	169.01	1.46	163.34	160.2	3.14
SC 1	148.2	145.98	2.22	157.06	154.86	2.2	158.03	154.49	3.54	166.25	163.69	2.56	173.07	170.52	2.55
2	155.62	152.41	3.21	162.61	160.74	1.87	163.24	159.92	3.32	164.4	163.24	1.16	178.55	176.38	2.17
3	135.15	131.71	3.44	142.18	139.76	2.42	143.25	138.88	4.37	156.91	154.58	2.33	145.29	142.07	3.22
4	141.44	137.78	3.66	140.83	138.04	2.79	148.95	144.38	4.57	152.1	149.17	2.93	162.4	158.81	3.59
5	137.63	135.14	2.49	139.17	136.6	2.57	153.48	149.53	3.95	148.55	146.61	1.94	146.55	144.23	2.32
6	154.58	152.21	2.37	155.93	153.7	2.23	173.79	170.9	2.89	175.25	172.4	2.85	160.25	156.77	3.48
7	138.75	135.92	2.83	138.3	134.27	4.03	151.97	147.38	4.59	150.6	147.8	2.8	152.39	148.77	3.62
8	136.84	134.15	2.69	136.12	132.82	3.3	145.87	142.13	3.74	158.47	154.94	3.53	160.7	157.18	3.52
SD 1	131.15	128.89	2.26	140.78	138.13	2.65	141.65	137.71	3.94	142.89	140.49	2.4	157.4	154.77	2.63
2	145.32	142.93	2.39	136.45	133.74	2.71	155.18	150.9	4.28	154.08	152.31	1.77	155.04	151.77	3.27
3	139.22	137.01	2.21	144.69	142.01	2.68	155.06	150.89	4.17	142.57	139.5	3.07	155.5	152.07	3.43
4	175.81	172.81	3	173.18	170.4	2.78	184.8	181.5	3.3	178.4	175.25	3.15	182.5	179.39	3.11
5	154.36	152.29	2.07	146.71	143.95	2.76	134.58	130.38	4.2	145.94	143.4	2.54	161.08	159.32	1.76
6	176.29	174.26	2.03	168.75	165.68	3.07	160.9	157.73	3.17	165.76	159.36	6.4	168.29	164.94	3.35
7	153.15	150.9	2.25	132.81	130.05	2.76	147.01	145.32	1.69	152.12	150.22	1.9	151.3	149	2.3
8	146.45	144.24	2.21	140.73	138.3	2.43	153.21	150.16	3.05	147.3	145.23	2.07	145.73	143.11	2.62
SE 1	144.23	139.77	4.46	142.02	139.4	2.62	161.29	157.54	3.75	135.59	132.76	2.83	180.69	176.36	4.33
2	169.98	168.07	1.91	169.85	168.43	1.42	172.03	169.34	2.69	171.26	169.44	1.82	171.5	169.2	2.3
3	144.91	142.34	2.57	141.77	139.51	2.26	161.92	159.26	2.66	149.54	147.4	2.14	136.36	133.73	2.63
4	149.09	146.16	2.93	142.38	139.54	2.84	151.04	147.11	3.93	138.47	136.14	2.33	145.01	143	2.01
5	151.4	148.6	2.8	139.77	138.05	1.72	144.15	140.76	3.39	141.27	138.73	2.54	147.39	144.22	3.17
6	163.07	161.4	1.67	160.17	157.45	2.72	181.44	179.78	1.66	162.54	160.05	2.49	158.06	155.6	2.46
7	142.21	139.9	2.31	133.85	130.37	3.48	148.35	144.78	3.57	136.9	134.35	2.55	152.87	149.63	3.24
8	137.68	134.78	2.9	135.41	131.88	3.53	159.1	155.48	3.62	142.25	139.49	2.76	145.5	142.28	3.22
SF1	142.15	140	2.15	141.51	139.5	2.01	147.49	144.19	3.3	138.63	136.2	2.43	154.89	152.2	2.69
2	149.19	147.42	1.77	139.62	136.39	3.23	156.58	153.22	3.36	139.31	136.1	3.21	145.03	141.81	3.22
3	147.78	145.9	1.88	139.65	137.16	2.49	154.36	152.31	2.05	143.25	141.01	2.24	149.78	147.15	2.63
4	135.04	132.69	2.35	139.01	136.5	2.51	136.81	133.64	3.17	141.2	138.6	2.6	151.5	148.99	2.51
5	154.58	152.85	1.73	151.39	148.87	2.52	145.94	143.62	2.32	156.75	153.68	3.07	149.99	146.49	3.5
6	149.91	147.61	2.3	148.37	145.71	2.66	155.67	152.63	3.04	148.13	145.6	2.53	155.13	151.85	3.28
7	145.41	143.37	2.04	138.44	135.93	2.51	141.47	138.19	3.28	142.05	140.3	1.75	139.8	137.34	2.46
8	150	148.05	1.95	154.39	151.06	3.33	152.06	149.15	2.91	151.43	149.21	2.22	167.94	164.86	3.08
SG 1	140.07	138.11	1.96	137.36	134.43	2.93	139.3	136.16	3.14	145.47	143	2.47	142.08	138.68	3.4
2	138.1	136.33	1.77	137.99	135.16	2.83	162.34	159.21	3.13	141.44	138.36	3.08	151.7	148.86	2.84
3	169.6	167.79	1.81	153	150.14	2.86	166.01	163.08	2.93	165.26	161.94	3.32	168.13	164.85	3.28
4	163.81	162.1	1.71	207.13	204.76	2.37	158.39	155.97	2.42	166.86	164.38	2.48	168.34	165.89	2.45
5	141.03	139.05	1.98	145.7	143.99	1.71	153.27	149.66	3.61	145.58	142.55	3.03	151.36	148.33	3.03
6	145.06	143.34	1.72	138.05	135.1	2.95	159.87	155.94	3.93	141.07	137.96	3.11	140.16	137.14	3.02
7	136.97	134.73	2.24	136.21	134.23	1.98	157.45	154.7	2.75	149.22	146.27	2.95	151.75	148.99	2.76
8	164.32	162.41	1.91	165.15	162.33	2.82	169.85	166.17	3.68	174.94	172.38	2.56	179.98	176.04	3.94
